# A SNAI2-PEAK1-INHBA stromal axis drives progression and lapatinib resistance in HER2-positive breast cancer by supporting subpopulations of tumor cells positive for antiapoptotic and stress signaling markers

**DOI:** 10.1038/s41388-021-01906-2

**Published:** 2021-07-08

**Authors:** Sarkis Hamalian, Robert Güth, Farhana Runa, Francesca Sanchez, Eric Vickers, Megan Agajanian, Justin Molnar, Tuan Nguyen, Joshua Gamez, Jonathan D. Humphries, Anupma Nayak, Martin J. Humphries, Julia Tchou, Ioannis K. Zervantonakis, Jonathan A. Kelber

**Affiliations:** 1 Department of Biology, California State University Northridge, Northridge, CA USA; 2 Wellcome Centre for Cell-Matrix Research, Faculty of Biology Medicine and Health, University of Manchester, Manchester, UK; 3 Department of Pathology and Laboratory Medicine, Hospital of the University of Pennsylvania and Perelman School of Medicine at University of Pennsylvania, Philadelphia, PA USA; 4 Division of Endocrine and Oncologic Surgery, Department of Surgery, Rena Rowan Breast Center, Abramson Cancer Center, Perelman School of Medicine at University of Pennsylvania, Philadelphia, PA USA; 5 Department of Bioengineering, University of Pittsburgh, Center for Bioengineering, Pittsburgh, PA USA; 6 Present Address: Department of Life Sciences, Manchester Metropolitan University, Manchester, UK

**Keywords:** Breast cancer, Cancer microenvironment, Cancer therapy, Mechanisms of disease, Prognostic markers

## Abstract

Intercellular mechanisms by which the stromal microenvironment contributes to solid tumor progression and targeted therapy resistance remain poorly understood, presenting significant clinical hurdles. PEAK1 (Pseudopodium-Enriched Atypical Kinase One) is an actin cytoskeleton- and focal adhesion-associated pseudokinase that promotes cell state plasticity and cancer metastasis by mediating growth factor-integrin signaling crosstalk. Here, we determined that stromal PEAK1 expression predicts poor outcomes in HER2-positive breast cancers high in SNAI2 expression and enriched for MSC content. Specifically, we identified that the fibroblastic stroma in HER2-positive breast cancer patient tissue stains positive for both nuclear SNAI2 and cytoplasmic PEAK1. Furthermore, mesenchymal stem cells (MSCs) and cancer-associated fibroblasts (CAFs) express high PEAK1 protein levels and potentiate tumorigenesis, lapatinib resistance and metastasis of HER2-positive breast cancer cells in a PEAK1-dependent manner. Analysis of PEAK1-dependent secreted factors from MSCs revealed INHBA/activin-A as a necessary factor in the conditioned media of PEAK1-expressing MSCs that promotes lapatinib resistance. Single-cell CycIF analysis of MSC-breast cancer cell co-cultures identified enrichment of p-Akt^high^/p-gH2AX^low^, MCL1^high^/p-gH2AX^low^ and GRP78^high^/VIM^high^ breast cancer cell subpopulations by the presence of PEAK1-expressing MSCs and lapatinib treatment. Bioinformatic analyses on a PEAK1-centric stroma-tumor cell gene set and follow-up immunostaining of co-cultures predict targeting antiapoptotic and stress pathways as a means to improve targeted therapy responses and patient outcomes in HER2-positive breast cancer and other stroma-rich malignancies. These data provide the first evidence that PEAK1 promotes tumorigenic phenotypes through a previously unrecognized SNAI2-PEAK1-INHBA stromal cell axis.

## Introduction

Cell state plasticity enhances intratumoral heterogeneity and has been shown to be a culprit underlying metastasis, therapy resistance and progression in cancer [1–4]. Previous studies have demonstrated a causative relationship between increased stromal tissue content (i.e., desmoplasia), including cancer-associated fibroblasts (CAFs) or mesenchymal stem cells (MSCs), in breast cancers and lapatinib resistance or metastasis [5–8]. In the case of HER2-positive breast cancer, where upregulation of the receptor tyrosine kinase HER2 (ErbB2) occurs in ~20% of all tumors [9], both trastuzumab- and lapatinib-based regimens offer significant clinical benefit [10]. However, a substantial percentage of these tumors display either primary resistance or may be initially sensitive but then adapt to develop acquired resistance [11], and clinical work suggests that patients who progress on lapatinib therapy commonly develop metastatic disease [12]. While recent work has reported that stromal fibroblasts limit HER2 kinase therapy responses via antiapoptotic signaling [13], the stromal cell non-autonomous mechanisms underlying HER2-targeted therapy resistance and/or resistance-associated metastasis remain poorly understood.

Pseudopodium-Enriched Atypical Kinase One (PEAK1 or SGK269) is a cytoskeleton-associated pseudokinase [14] and member of the new NKF3 kinase family that has been demonstrated to play key cancer cell autonomous roles in cancer initiation and progression across multiple cancer types including breast [15–17], pancreatic [14, 18], lung [19] and colon [14, 20, 21]. We previously reported that PEAK1 functions downstream of eIF5A1/2-dependent translation in mediating epithelial-mesenchymal transition (EMT), metastasis and transforming growth factor beta (TGFβ)/fibronectin signaling [15–17, 22]. In this regard, PEAK1 has been identified as part of the meta-adhesome [23] and core constituent of the fibroblast adhesome [24]. Zheng and colleagues reported that PEAK1 is a critical adapter protein governing Shc1 association with cytoskeletal reorganization, trafficking and signal termination proteins downstream of EGF/Akt/PTPN12 activity to mediate cell invasion [25].

Here, we address whether PEAK1 may promote tumorigenesis via the non-epithelial stromal compartment of solid tumors. To this end, we report that PEAK1 expression in breast cancer stroma is associated with relapse in HER2-positive breast cancer and that PEAK1 is predominantly expressed in tumor associated SNAI2-positive fibroblast-like cells. In agreement with these data, patient-derived CAFs and MSCs express PEAK1 and can promote malignant phenotypes and lapatinib resistance in vitro and in vivo in a PEAK1-dependent manner. Finally, we combine protein array and single-cell CycIF multiplex methods to identify a previously unrecognized PEAK1-INHBA-antiapoptotic stromal-tumor cell signaling axis that may be leveraged to abrogate therapeutic resistance in HER2-positive breast cancer and improve patient outcomes.

## Materials and methods

### Cell culture

Cell origin and culture method details are described in the Supplementary Materials and Methods.

### Bioinformatics

Data mining and analyses procedures are described in the Supplementary Materials and Methods.

### Immunohistochemistry

Formalin-fixed tissue samples were sent to the UCLA Tissue Procurement Core Laboratory for paraffin embedding, tissue sectioning and H&E staining. Alternatively, primary breast cancer tissue was obtained as single tumor sections from Dr. Julia Tchou or purchased as a tissue microarray (TMA) from USBiomax. Details on antibody, staining and imaging procedures are described in the Supplementary Materials and Methods.

### Conditioned media (CM)

Cells were plated in 10 cm plates at 6e5 cells and incubated until 70% confluent. Media in each 10 cm plate was changed to 6 mL of appropriate media without serum and incubated for an additional 48 h. Mock/control media was made by placing the same media into a plate without cells for 48 h. Media was collected, centrifuged at 1000 rpm for 5 min and used right away or stored at −80 °C in 15 mL aliquots until needed. Before the CM was used in experiments, it was diluted 1:1 with the appropriate fresh serum-free media. Subsequent ELISA analyses on CM for activin-A was performed using the activin-A DuoSet ELISA (R&D Systems) in accordance with manufacturers instructions.

### Western blot

Cell extract collection and immunoblot reagents and procedures are described in the Supplementary Materials and Methods.

### Immunocytochemistry

Details on antibody, staining and imaging procedures are described in the Supplementary Materials and Methods.

### Chorioallantoic membrane assay (CAM assay)

Animal origin and use procedures are described in the Supplementary Materials and Methods. All procedures were completed in accordance with IACUC protocol # 1920-008b. qPCR analysis details are also described within the Supplementary Materials and Methods.

### Lentiviral transduction

Cells were plated at 4.8e6 cells/well into a 6 well plate and left to attach overnight. Viral mixes were created with an aliquot of virus into complete media and polybrene at 8μg/ml (Sigma-Aldrich) to have a target multiplicity of infection (MOI) of 5. Viral particles contained a puromycin resistant pKLO.1 vector with a scramble shRNA or PEAK1-specific shRNA (5 different constructs). Viral mixes were added to their respective wells and left to incubate for 24 h, after which regular media was replaced. The following day, media was changed and supplemented with 1 ug/mL puromycin. Cells were expanded and knockdown efficacy was validated by Western blotting.

### Cell proliferation/viability assay

Cell proliferation/viability was measured using the CellTiter 96® AQueous One Solution (Promega). Cell plating and analysis procedures are described in the Supplementary Materials and Methods.

### IncuCyte

IncuCyte® Live Cell Analysis Imaging System was used according to manufacturer’s protocol. Cell plating and analysis procedures are described in the Supplementary Materials and Methods.

### Protein microarray

The semi-quantitative RayBio L-series mouse antibody array L-308 was used according to manufacturer protocol for analysis of cell lysates from the C3H10T1/2 shRNA derivatives. Processed slides were imaged and analyzed using a GenePix 400B instrument and Molecular Devices software.

### Cyclic immunofluorescence (CycIF)

Cyclic multiplex antigen staining and Hoescht nuclear counterstaining was carried out on paraformaldehyde (PFA)-fixed mono- or co-culture cells on ultra-optically clear, flat-bottom, black-walled 96-well plates as previously described [26]. Cell plating, reagent, staining and analysis procedures are described in the Supplementary Materials and Methods.

### Statistics

All quantified data were plotted and analyzed in GraphPad Prism with ANOVA, Student’s *t* test, or nonlinear regression analysis. Data reported are representative of at least 3 independent biological replicates and are reported as technical replicate averages ± SEM, unless otherwise indicated. *, **, *** or **** represent *p* values < 0.05, 0.01, 0.001, or 0.0001 respectively, unless otherwise noted.

## Results

### A SNAI2-PEAK1 stromal axis correlates with disease progression in HER2-positive breast cancer

**Fig. 1:**
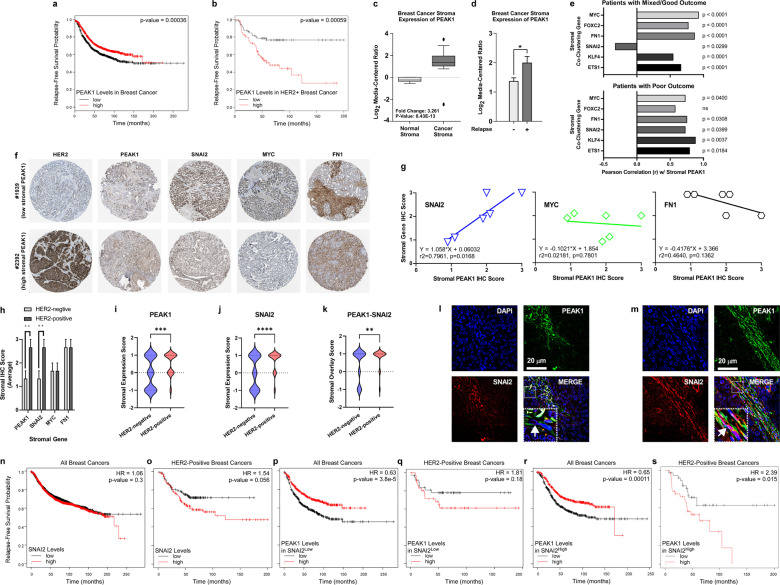
A SNAI2-PEAK1 axis correlates with disease relapse and co-stains the non-epithelial fibroblastic stroma in HER2-positive breast cancer. **a** Kaplan–Meier relapse-free survival (RFS) curves for patients with low or high PEAK1 transcript levels across all breast cancer subtypes (*n* = 1784). **b** Kaplan–Meier RFS curves for HER2-positive breast cancer patients with low or high PEAK1 transcript levels (*n* = 272). **c** PEAK1 transcript levels in normal breast stroma and breast cancer stroma across all subtypes (*n* = 6 and 53, respectively). **d** PEAK1 transcript levels in breast cancer stroma of relapse-free patients and those with disease recurrence across all subtypes (*n* = 42 and 11, respectively). **e** Expression correlation analysis of PEAK1 and ETS1, KLF4, SNAI2, FN1, FOXC2 or MYC in patients having mixed or good outcomes (top, *n* = 45) and patients having poor outcomes (bottom, *n* = 8). **f** Representative IHC images for HER2, PEAK1, SNAI2, MYC and FN1 in breast cancer tissue where PEAK1 expression in the stromal compartment is low (top) or high (bottom). **g** Stromal IHC score correlation and linear regression analyses of PEAK1 and SNAI2, MYC or FN1 across six breast cancer tissue samples (patient #s 1874, 1939, 2428, 1920, 2392 and 3257). **h** Average stromal IHC scores for PEAK1, SNAI2, MYC and FN1 in three HER2-negative patients and three HER2-positive patients. **i**–**k** Violin plots of quantified PEAK1, SNAI2 or PEAK1/SNAI2 stromal expression from IHC/IF data on a 144-breast cancer sample tissue microarray (79 HER2-positive cases). **j**–**m** Representative 3D deconvolution widefield microscopy images for PEAK1 and SNAI2 in TB130 (HER2-negative) and TB122 (HER2-positive) breast cancer tissues. **n**–**o** Kaplan–Meier relapse-free survival (RFS) curves for patients with low or high SNAI2 in all breast cancer subtypes (**n**, *n* = 1784) or HER2-positive breast cancer (**o**, *n* = 272). Kaplan–Meier relapse-free survival (RFS) curves for patients with low or high PEAK1 in all SNAI2^low^ or SNAI2^high^ breast cancers (**p** and **r**, *n* = 892) and HER2-positive SNAI2^low^ or SNAI2^high^ breast cancers (**q** and **s**, *n* = 136). *, **, *** and **** indicates *p* value < 0.05, 0.01, 0.001 and 0.0001, respectively, as determined by a Student’s *T* test.

We first examined patient survival across all breast cancer subtypes in relation to PEAK1 expression levels. The KMPlot resource enabled assessment of relapse-free survival (RFS), distant metastasis-free survival (DMFS) and overall survival (OS) across more than 3000 patients [27, 28]. Elevated PEAK1 expression across all breast cancer subtypes predicted a significant, though slight, increase in RFS (Fig. 1a), while elevated PEAK1 expression in HER2-positive breast cancers correlated with decreased RFS suggesting a role for PEAK1 in this more aggressive breast cancer subtype (Fig. 1b). In contrast, elevated PEAK1 expression alone across all breast cancer subtypes had a very modest prognostic association with OS or DMFS (Supplementary Fig. 1). In parallel, we mined data [29] on breast cancer stromal gene expression and discovered that PEAK1 expression was significantly higher in malignant breast stroma (Fig. 1c), and that elevated stromal PEAK1 expression positively correlated with disease relapse (Fig. 1d).

To identify breast cancer stromal gene networks associated with increased PEAK1 expression and poor outcome, we analyzed the relationships between expression patterns for gene signatures corresponding to epithelial (9 genes), mesenchymal (19 genes), stem (4 genes) and mesenchymal stem (15 genes) markers. These signatures were clustered relative to PEAK1 in stromal tissue samples across patient groups previously classified as having poor, mixed or good outcomes [29] (Supplementary Fig. 2a). Expression correlation analysis identified six genes (i.e., ETS1, KLF4, SNAI2, FN1, FOXC2 and MYC) that strongly clustered with PEAK1 in the poor outcome group. Notably, the SNAI2-PEAK1 relationship shifted from a significant negative correlation in patients having mixed/good outcomes to a significant positive correlation across patients having poor outcomes (Fig. 1e). In further support of a SNAI2-PEAK1 stromal cell signaling axis in breast cancer, we noted a significant positive correlation between SNAI2 and PEAK1 protein levels in the stromal compartment of breast cancer samples (Fig. 1f, g), and that these SNAI2 and PEAK1 protein levels were specifically expressed at higher levels within the stroma of HER2-positive tumors (Fig. 1h). We further stained tissue microarrays (TMAs) for PEAK1, SNAI2 and CDH1 and analyzed the PEAK1/SNAI2 expression/co-expression patterns within the CDH1-negative stromal compartment. This revealed that the stromal co-expression of PEAK1 and SNAI2 was significantly increased in HER2-positive tumor tissues (*n* = 79) (Fig. 1i–k and Supplementary Fig. 2b). Additional patient sample staining revealed further that the cytoplasmic PEAK1 and nuclear SNAI2 expression within the stromal tissue of HER2-positive breast cancers can occur within the same fibroblastic cell types – a pattern not observed in the HER2-negative patient samples (Fig. 1l, m). Like PEAK1, elevated SNAI2 expression in HER2-positive breast cancer predicts reduced RFS (Fig. 1n, o) as well as DMFS (Supplementary Fig. 3a) in HER2-positive breast cancer – patterns also observed for fibronectin in this same cancer subset (Supplementary Fig. 3c). In support of a cooperative role for PEAK1 and SNAI2 within the same fibroblastic stromal cells in HER2-positive breast cancers, the poor prognostic utility of PEAK1 in HER2-positive breast cancer was notably restricted to patient tumors expressing high levels of SNAI2 (Fig. 1p–s and Supplementary Fig. 3).

### SNAI2 and PEAK1 coexpression in breast cancers enriched for mesenchymal stem cell content is prognostically unfavorable

**Fig. 2:**
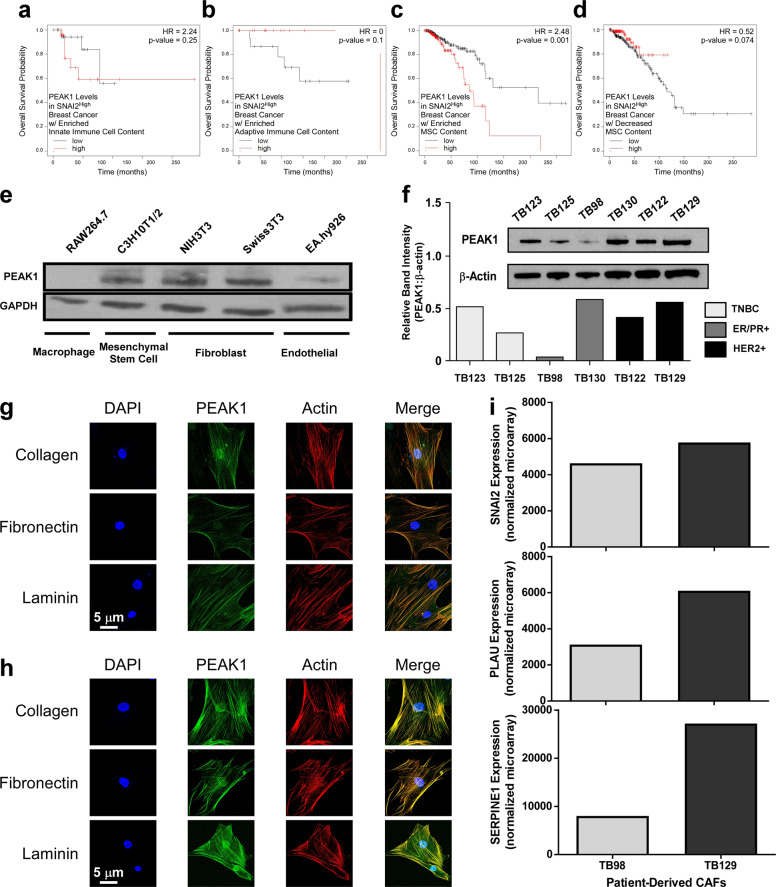
SNAI2 and PEAK1 coexpression in breast cancers enriched for mesenchymal stem cell content is prognostically unfavorable. **a**–**c**. Kaplan–Meier OS curves for low or high PEAK1 transcript levels in breast cancer patients selected for high SNAI2 expression and enriched innate immune (**a**), adaptive immune (**b**) or mesenchymal stem cell contents (*n* = 45, 19 and 382, respectively). **d** Kaplan–Meier OS curves for low or high PEAK1 transcript levels in breast cancer patients selected for high SNAI2 expression and decreased mesenchymal stem cell content (*n* = 381). **e** Western blot for PEAK1 and GAPDH in total lysates from the indicated non-tumorigenic cell lines. **f** Western blot and relative band intensity for PEAK1 and β-actin in total lysates from the indicated patient-derived breast cancer-associated fibroblasts. Representative confocal microscopy images for nucleus (DAPI), filamentous actin (Phalloidin) and PEAK1 in TB98 (**g**) and TB129 (**h**) CAF lines plated onto 5 ug/mL collagen, fibronectin or laminin substrates. **i** Normalized expression of SNAI2, PLAU and SERPINE1 in the indicated TB CAF lines obtained from the GEO database using the GSE37614 dataset.

We next mined clinical data for relationships between high coexpression of SNAI2 and PEAK1 and OS across patient tissues enriched for specific stromal cell types. High expression of both SNAI2 and PEAK1 did not predict OS probability among breast cancer patients reporting enrichments in either innate or adaptive immune cell content (Fig. 2a, b). However, high expression of both SNAI2 and PEAK1 predicted significantly lower OS among patients with mesenchymal stem cell (MSC) content (Fig. 2c). In contrast, high PEAK1 expression levels among patients with high SNAI2 expression was not prognostically significant in patients with decreased MSC content (Fig. 2d).

By evaluating PEAK1 expression across non-tumor cell types (i.e., 3 fibroblast-like, 1 endothelial and 1 innate immune cell lines), we further established that PEAK1 expression was highest within fibroblast-like cell types (Fig. 2e). These data were further supported by analyzing the expression of PEAK1 across a subset of patient-derived cancer-associated fibroblasts (CAFs) (i.e., two from each breast cancer subtype) previously isolated and transcriptomically profiled [30] (Fig. 2f). At the subcellular level, PEAK1 localized strongly with the actin cytoskeleton in both the TB98 (ER-positive subtype) and TB129 (HER2-positive subtype) CAFs independent of extracellular matrix (ECM) substrate (Fig. 2g, h and Supplementary Fig. 4). Finally, analysis of SNAI2 and two other mesenchymal stromal cell genes (i.e., PLAU and SERPINE1) revealed their collective expression to be higher in the TB129 CAF line relative to the TB98 line (Fig. 2i).

### Chicken embryo chorioallantoic membrane (CAM) xenografting of patient-derived CAFs or MSCs with HER2-positive breast cancer cells increases primary tumor mass

**Fig. 3:**
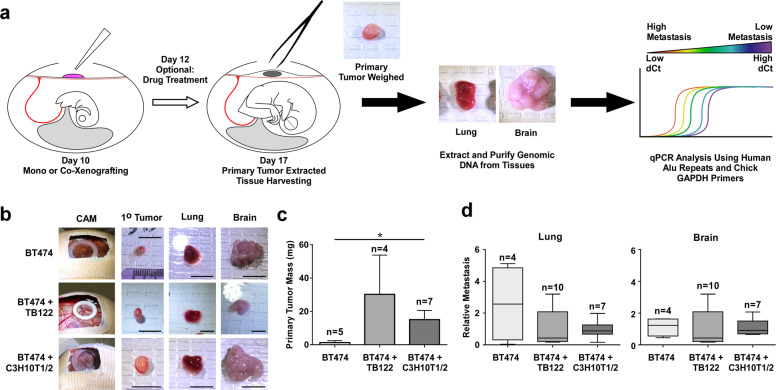
Chicken embryo chorioallantoic membrane (CAM) xenografting of patient-derived CAFs or MSCs with HER2-positive breast cancer cells increases primary tumor mass. **a** Schematic of the chorioallantoic membrane (CAM) xenograft system using chicken (*Gallus gallus*) embryos together with human tumor cells and end point analysis of whole tissue genomic DNA by qPCR for human alu repeats and host chicken Gapdh levels. The method has been modified from the original assay system [32] to enable a 5-day drug treatment regimen beginning at 2 days post-xenograft. **b** Representative images of BT474 cells, BT474 cells + TB122 CAFs or BT474 cells + C3H10T1/2 mesenchymal stem cells. Scale bar = 1 cm. **c** Quantified primary tumor mass of experiments represented in **b**. **d** Relative metastasis of BT474 cells in the lung (left) and brain (right) of experiment in **b**. *Indicates a *p* value < 0.05 as determined by a One-Way ANOVA w/ multiple comparisons post-test.

We next asked whether PEAK1-expressing CAFs or MSCs could affect breast tumor growth and progression in the *Gallus gallus* embryo chorioallantoic membrane (CAM) in vivo model [31–33] (Fig. 3a). As shown in Fig. 3b, c, the mass of BT474-derived tumors significantly greater when xenografted together with either the TB122 CAFs or C3H10T1/2 MSCs, although neither the CAF- nor MSC-containing xenografts displayed a measurable difference in early metastatic dissemination events to lung or brain tissues (Fig. 3d). Similarly, MCF7 cells xenografted together with TB130 CAFs or together with TB130 CAFs after in vitro pre-incubation with TB130 CAF conditioned media (CM) formed larger primary tumors while differences in early metastatic dissemination events to the lung and brain tissue were not observed (Supplementary Fig. 5). These results support using this system, in agreement with previous reports [34–36], to interrogate the role of PEAK1 in MSC-mediated HER2-positive breast cancer progression and targeted therapy response.

### Knockdown of PEAK1 in MSCs abrogates their ability to promote tumorigenesis, intratumoral αSMA expression, lapatinib resistance and lapatinib-induced brain metastasis

**Fig. 4:**
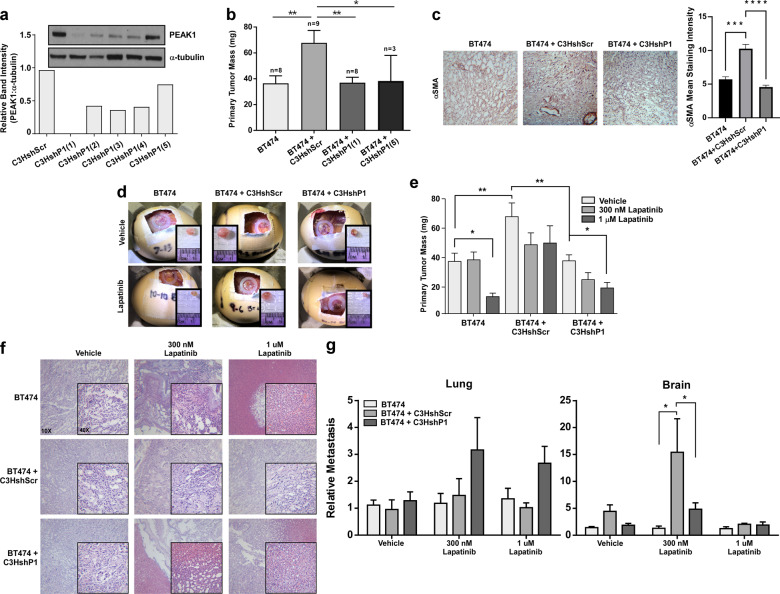
Knockdown of PEAK1 in MSCs abrogates their ability to promote tumorigenesis, intratumoral αSMA expression, lapatinib resistance and lapatinib-induced brain metastasis. **a** Western blot and relative band intensity quantification for PEAK1 and α-tubulin levels in shScramble control (shScr) and 5 different PEAK1-targeting (shP1) shRNA derivatives of C3H10T1/2 mesenchymal stem cells. Unless otherwise noted, shP1(1) construct is used throughout experiments. **b** Quantified primary tumor mass of CAM xenograft assay using BT474 cells only or BT474 cells xenografted together with the C3HshScr, C3HshP1(1) or C3HshP1(5) cells. **c** Representative images and quantification of alpha-smooth muscle actin staining of stromal tissue in CAM tumors from **b**. **d** Representative assay endpoint images for CAM xenograft experiment using the same cell combinations as in **b** with either vehicle control or 1 uM lapatinib treatments. **e** Quantified primary tumor mass of experiment in **d** including the treatment condition of 300 nM lapatinib. **f** Hematoxylin and eosin staining of CAM tumor tissue in **d**. **g** Relative metastasis of BT474 cells in the lung (left) and brain (right) of experiment in **d**. *, **, *** and **** indicates a *p* value < 0.05, 0.01, 0.001 and 0.0001, respectively, as determined by a One-Way or Two-Way ANOVA w/ multiple comparisons post-test.

A panel of stable shRNA C3H10T1/2 MSC derivatives containing either a scramble control shRNA construct (shScr) or one of five PEAK1-targeting shRNAs (shP1) was generated (Fig. 4a). As before with the parental C3H10T1/2 MSCs, xenografting the shScr MSCs with the BT474 cells significantly increased primary tumor mass – an effect that was abrogated by PEAK1 knockdown using two unique shRNA constructs (Fig. 4b). Notably, PEAK1-expressing MSCs caused BT474 tumors to contain elevated alpha-smooth muscle actin (αSMA) staining and vascular-like structures (Fig. 4c).

We then analyzed whether these MSCs could render BT474 tumors resistant to lapatinib treatment in vivo and whether any observed effects might require PEAK1 expression. Notably, shScr MSCs rendered BT474 cells less sensitive to lapatinib in vivo. Furthermore, BT474 xenografts containing MSCs with the PEAK1-targeting shRNAs responded to lapatinib as though there were no MSCs xenografted (Figs. 4d, e). In agreement with these data, hematoxylin and eosin (H&E) staining revealed a high degree of necrotic tissue around the periphery of tumors generated from only BT474 cells alone or BT474 xenografts containing MSCs with the PEAK1-targeting shRNAs at both lapatinib doses (Fig. 4f). Interestingly, the presence of PEAK1-expressing MSCs in primary tumors treated with intermediate doses of lapatinib enabled the BT474 cells to escape and metastasize to the brain at a 15-fold greater frequency when compared to xenografts of the BT474 cells alone or BT474 cells and shP1 MSCs (Fig. 4g).

### MSC expression of PEAK1 protects neighboring breast cancer cells from lapatinib-induced cytotoxicity

**Fig. 5:**
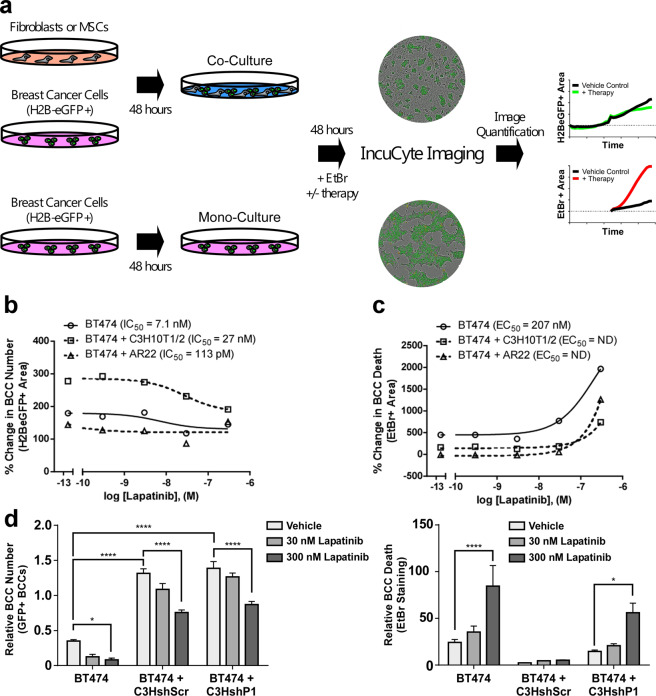
MSC expression of PEAK1 protects neighboring breast cancer cells from lapatinib-induced cytotoxicity. **a** Schematic of breast cancer cell mono- or breast cancer cell-CAF/MSC co-culture using non-labeled stromal fibroblasts and H2B-eGFP+ BT474 breast cancer cells for downstream analysis of breast cancer cell number and death using the IncuCyte imaging system over 96 h (48 h pre-incubation and 48 h incubation with therapy and EtBr). **b**–**c** Endpoint dose-response curves for lapatinib effects on breast cancer cell number (**b**) or cell death (**c**) in the indicated breast cancer cell and stromal fibroblast culture combinations. **d** Quantification of tumor cell number (left) and EtBr uptake (right) at assay endpoint for BT474 cells alone or co-cultured with the shScr or shP1(5) derivatives of C3H10T1/2 cells and treated with vehicle control or the indicated dose of lapatinib. * of **** indicates a *p* valule of 0.05 or 0.0001, respectively as determined by a Two-Way ANOVA with multiple comparisons post-test.

To elucidate potential mechanisms by which these stromal cells elicit their tumor- and lapatinib resistance-promoting functions, we employed co-culture methods [13] to further evaluate whether MSCs or breast fibroblasts could promote breast cancer cell expansion and resistance to lapatinib in vitro (Fig. 5a). Co-seeding either MSCs or AR22 breast fibroblasts together with H2B-eGFP labeled BT474 cells established monolayer co-cultures in which the breast cancer cells formed islands surrounded by fibroblasts (Fig. 5a and Supplementary Fig. 4a). We then used this system in combination with IncuCyte imaging to evaluate both the number of eGFP-positive and EtBr-positive breast cancer cells during time-course lapatinib dose-response experiments (Fig. 5a and Supplementary Fig. 6a). As shown in Supplementary Fig. 6b, mono-cultures of either the MSCs or fibroblasts did not respond to increasing lapatinib doses as measured by EtBr uptake while the BT474 mono-cultures did, demonstrating that this targeted therapy displays specific cytotoxicity to HER2-overexpressing breast cancer cells. Interestingly, while co-culture of BT474 cells together with MSCs was able to both increase the basal number of BT474 cells (Fig. 5b) and reduce lapatinib cytotoxicity (Fig. 5c), the breast fibroblasts seemed to selectively reduce lapatinib cytotoxicity (Fig. 5c and Supplementary Fig. 6b). Using the shScr and shP1 MSC derivatives in this co-culture assay revealed that PEAK1 expression mediates the ability for MSCs to protect neighboring breast cancer cells against lapatinib-induced cytotoxicity (Fig. 5d).

### PEAK1 expression in MSCs drives the production of secreted factors that promote breast cancer cell proliferation/survival and lapatinib resistance in vitro

**Fig. 6:**
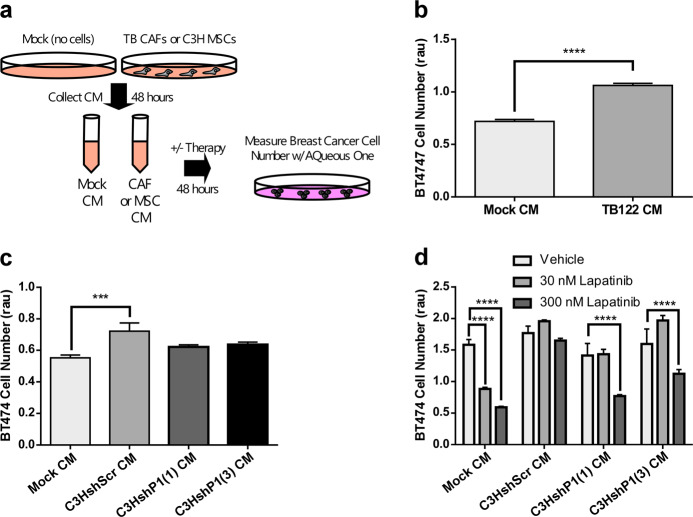
PEAK1 expression in MSCs drives the production of secreted factors that promote breast cancer cell proliferation/survival and lapatinib resistance in vitro. **a** Schematic for generating TB CAF or C3H MSC conditioned media (CM) for analysis on breast cancer cell growth/survival over 48 h in vitro. **b** Cell viability analysis of BT474 cells treated with mock or TB122 CM. **c** Cell viability analysis of BT474 cells treated with mock CM or CM from the indicated shRNA derivatives of C3H10T1/2 cells. **d** Cell viability analysis of BT474 cells treated with mock CM or CM from the indicated shRNA derivatives of C3H10T1/2 cells and treated with vehicle control or the indicated dose of lapatinib. *, ***, or **** indicates a *p* value < 0.05, 0.001 or 0.0001, respectively, as determined by a One-Way or Two-Way ANOVA w/multiple comparisons post-test.

We also tested conditioned media (CM) from these cell types for their ability to promote breast cancer cell expansion and/or lapatinib resistance in vitro (Fig. 6a). CM collected from CAFs derived from either HER2-positive (Fig. 6b) or ER-positive (Supplementary Fig. 7a) breast cancers potentiated BT474 or MCF7 cell growth, respectively. Notably, by using shRNA MSC derivatives, PEAK1 was found to be necessary to produce secreted factors into MSC CM that potentiate BT474 (Fig. 6c) or mouse Py230 cell growth (Supplementary Fig. 7b). Finally, MSC expression of PEAK1 was necessary for MSC-derived CM to promote BT474 cell resistance to lapatinib (Fig. 6d).

### PEAK1-dependent INHBA/activin-A expression/secretion from MSCs mediates MSC-induced lapatinib resistance in HER2-positive breast cancer cells

**Fig. 7:**
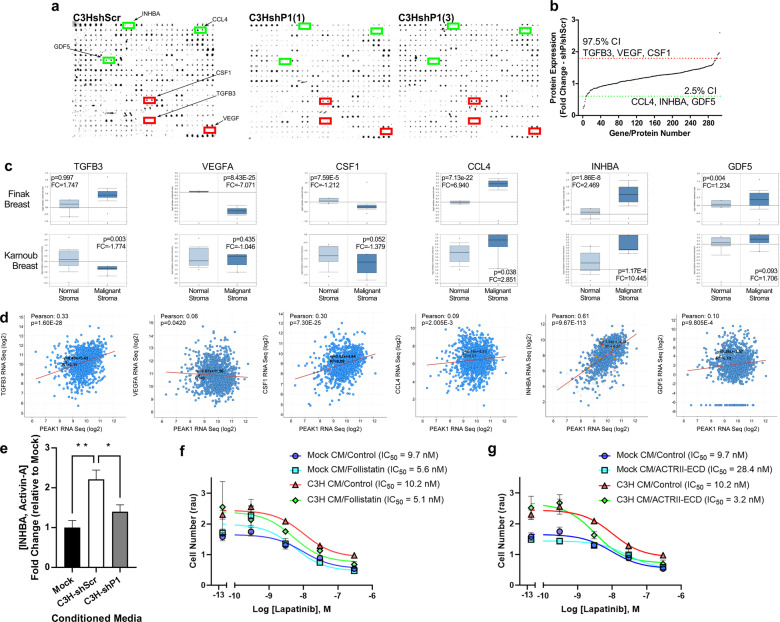
PEAK1-dependent INHBA/activin-A expression/secretion from MSCs mediates MSC-induced lapatinib resistance in HER2-positive breast cancer cells. **a** Representative slide scan images from the semi-quantitative mouse antibody array 308 (L-308) following incubation and reactivity with total cell lysates prepared from the shScramble and indicated PEAK1-specific shRNA derivatives of the C3H10T1/2 MSCs. **b** Quantification of ranked protein expression across the 308 array antigens with confidence intervals set to identify antigen expression changes up (red line) and down (green) with a *p* value < 0.05. **c** Relative mRNA expression for the indicated PEAK1-dependent cytokines in normal versus malignant stroma as reported in the indicated studies. **d** Expression relationship for TGFB3, VEGFA, CSF1, CCL4, INHBA and GDF5 versus PEAK1 mRNA levels in breast cancer patients. **e** ELISA analysis for activin-A in mock conditioned media or media conditioned with the indicated shRNA derivatives of the C3H10T1/2 cells. **f**–**g** Aqueous One cell viability assay on BT474 cells treated with either mock or C3H10T1/2 cell conditioned media and increasing doses of lapatinib in the presence of control or 1 μg/mL Follistatin (**f**) or ACTRII-ECD (**g**).

To identify the factors that PEAK1 regulates within MSCs, we performed semi-quantitative protein array analysis targeting 308 protein antigens in lysates from the shScr and two unique shP1 MSC derivatives (Fig. 7a). PEAK1 knockdown led to a greater than 2-fold decrease in 5 proteins (GDF5, CCR4, INHBA/activin-A, GRH and CCL4) and a greater than 1.8-fold increase in 7 additional proteins (PDGFRA, CSF1, HGFR, Frizzled-6, VEGFA, PF4 and TGFB3) (Dataset 1). As shown in Fig. 7b, six PEAK1-dependent soluble factors met the 95% confidence interval cut-off criteria for further analysis. We next sought to determine whether there was any clinical relevance of these PEAK1-regulated MSC gene/proteins (i.e., TGFB3, VEGFA, CSF1, CCL4, INHBA and GDF5). As with PEAK1 (Fig. 1), we analyzed expression profiles for these genes across two independent studies investigating normal breast stroma and malignant breast stroma [6, 29]. Notably, the transcripts of PEAK1-suppressed MSC factors (i.e., TGFB3, VEGFA and CSF1) were significantly lower within breast cancer stroma (Fig. 7c, left three graphs). In contrast, the factors whose expression was dependent upon PEAK1 in MSCs (i.e., CCL4, INHBA and GDF5) displayed significantly higher transcript levels in breast cancer stroma across both studies, with INHBA/activin-A showing the greatest average fold-change increase in malignant over normal breast stroma (Fig. 7c, right three graphs). Analysis of the mRNA expression relationship between PEAK1 and each of these six genes in breast cancer patient tissues [37] revealed that PEAK1 and INHBA transcripts showed the most significant positive correlation (Fig. 7d). Additional analysis of RFS and OS based upon elevated expression for each of these six factors in HER2-positive together with PEAK1 or an enriched MSC signature (Supplementary Fig. 8a–c) highlighted the prognostic importance for INHBA/activin-A. ELISA analysis was performed on mock media or conditioned medias from the shRNA MSC derivatives and revealed that soluble activin-A was only detectable in the conditioned media from PEAK1-expressing C3H MSCs (Fig. 7e). Finally, using two unique activin-A antagonists (i.e., Follistatin and ACTRII-ECD), we demonstrate that the lapatinib protective effects of MSC conditioned media requires activin-A (Fig. 7f, g).

### PEAK1-expressing MSCs promote lapitinib resistance by modulating antiapopototic/DNA damage signaling within a subpopulation of highly plastic HER2-positive breast cancer cells

**Fig. 8:**
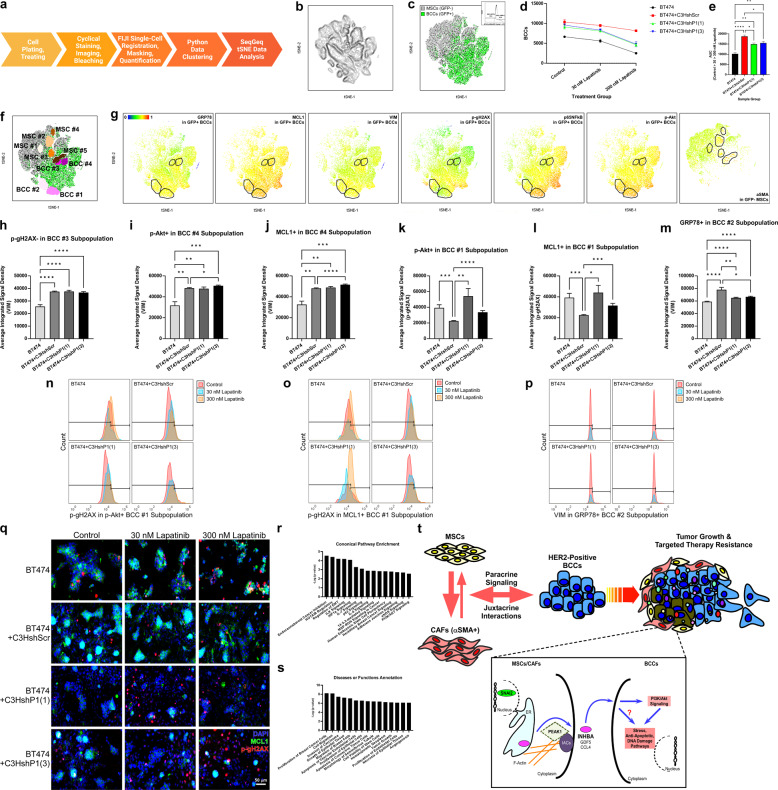
PEAK1-expressing MSCs promote lapitinib resistance by modulating antiapopototic/DNA damage signaling within a subpopulation of highly plastic HER2-positive breast cancer cells. **a** Schematic of the CycIF workflow used for single-cell analysis. **b**–**c** t-SNE plots of all cells across biological replicates of cell culture and lapatinib treatment conditions (**b**) and overlayed with the GFP-positive BT474 HER2-positive breast cancer cells (**c**, inlay shows GFP gating scheme). **d** Averaged BT474 GFP-positive breast cancer cell (BCC) number at 48 h post-therapy treatment. **e** Quantification of ar–––ea under the curve (AUC) for data plotted in **d**. **f** Overlay of notable breast cancer cell and MSC subpopulations onto the t-SNE plot from **c**. **g** Pseudocoloring of the single-cell antigen intensities of GRP78, MCL1, VIM, p-γH2AX, p65NFκB and p-Akt in the GFP-positive breast cancer cells and αSMA in the GFP-negative MSCs overlayed onto the t-SNE plots for these cell populations. **h**–**m** Average integrated signal intensity for indicated tumor cell markers within indicated gated populations of complementary markers across the four cell culture conditions. **n**–**p** Histograms representing lapatinib-induced changes of the single-cell antigen expression patterns for p-Akt^high^/p-gH2AX^low^, MCL1^high^/p-gH2AX^low^ and GRP78^high^/VIM^high^ BT474 cell subpopulations identified in **k**–**m**. **q** Representative microscopy images of nuclear (DAPI), MCL1 and p-gH2AX immunofluorescence across cell culture and lapatinib treatment conditions. IPA-derived canonical pathway (**r**) or disease/function annotation enrichments (**s**) for SNAI2, PEAK1, INHBA, CCL4, GDF5, MCL1, AKT1, H2AFX, GRP78 and VIM. **t** Proposed model of mechanism by which stromal expression of PEAK1 drives tumor growth, metastasis and targeted therapy resistance in neighboring HER2-positive breast cancer cells.

To capture HER2-positive tumor cell states changes in the presence of PEAK1-expressing MSCs and in response to lapatinib treatment, we performed single-cell Cyclic Immunofluorescence (CycIF) as previously described [26] in combination with the co-culture system described in Fig. 5a (Fig. 8a) across seven unique cell state markers measuring apoptosis evasion (i.e., MCL1), growth signaling (i.e., p-Akt), metastasis (i.e., VIM), DNA damage (i.e., p-γH2AX), oxidative response (i.e., p65NFκB), stress response (i.e., GRP78) and microenvironment fibroblast activation (i.e., αSMA). Single-cell quantification of these antigen markers across three biological replicates for four cell culture conditions and three lapatinib treatment conditions produced 3,462,844 data points in 494,692 single cell events. T-distributed stochastic neighbor embedding (t-SNE) was used to reduce data dimensionality (Fig. 8b) and to gate on GFP-positive breast cancer cells (Fig. 8c). This analysis demonstrated good cell resolution and lapatinib-induced reduction in breast cancer cell number that was significantly blocked by the presence of PEAK1-expressing MSCs (Fig. 8d, e). Inspection of the t-SNE outputs for BT474 cell monocultures or the BT474 + C3HshRNA cocultures across control, 30 nM or 300 nM lapatinib treatment conditions (Supplementary Fig. 9a) allowed us to identify four breast cancer cell and five MSC subpopulations that emerged in BT474 + C3HshScr cocultures and persisted in the presence of lapatinib (Fig. 8f). p-Akt, p65NFκB, p-γH2AX, MCL1, GRP78 and VIM markers were expressed highest in the GFP-positive breast cancers while αSMA was expressed predominantly in the GFP-negative MSCs (Fig. 8g). In agreement with our in vivo data (Fig. 4c), we observed a striking PEAK1-dependent increase in αSMA expression across all populations of MSCs in co-culture with BT474 cells (Supplementary Fig. 9b).

Systematic analysis of the antigen integrated signal densities in GFP-positive breast cancer cells initially gated for positive/negative expression of each of the other antigen markers (Supplementary Fig. 9c–f) revealed three unique subpopulations of breast cancer cells enriched in the presence of MSCs (i.e., p65NFκB^high^/VIM^high^, p-Akt^high^/VIM^high^ and MCL1^high^/VIM^high^) that were not dependent upon MSC expression of PEAK1 (Fig. 8h–j) and three unique subpopulations enriched in the presence of only PEAK1-expressing MSCs (i.e., p-Akt^high^/p-γH2AX^low^, MCL1^high^/p-γH2AX^low^ and GRP78^high^/VIM^high^) (Fig. 8k–m). Notably, these p-Akt^high^/p-γH2AX^low^, MCL1^high^/p-γH2AX^low^ and GRP78^high^/VIM^high^ subpopulations persisted in the presence of lapatinib treatment (Fig. 8n–p). In agreement with these data, immunofluorescence for MCL1 and p-γH2AX across the four cell culture conditions demonstrate that co-culture of BT474 cells with PEAK1-expressing MSCs encircled MCL1^high^/p-gH2AX^low^ cells in the presence of lapatinib (Fig. 8q)

Finally, we used Ingenuity Pathway Analysis (IPA) to evaluate the pathway, disease and functional annotations for a 10 gene set comprised of PEAK1, PEAK1-associated/dependent genes from the mesenchymal tumor stroma (i.e., SNAI2, INHBA, CCL4 and GDF5) and markers identified in our CycIF screen to be enriched in HER2-positive breast cancer cells when exposed to PEAK1-expressing MSC (i.e., AKT1, H2AFX, MCL1, GRP78 and VIM) (Fig. 8r–s). Expanded interactome analysis of these core genes using Cytoscape, generated 9 subnetworks that included enriched gene ontologies for regulation of transcription, antiapoptosis, mesoderm morphogenesis, stress responses, DNA damage responses, mesenchymal cell survival, exctracellular matrix (ECM) disassembly and JAK-STAT signaling (Supplementary Fig. 10). Taken together, we identify a pharmacologically targetable PEAK1-INHBA-dependent and SNAI2-associated stromal cell non-autonomous mechanism through which neighboring HER2-positive breast cancer cells increase mitogenic, antiapoptotic and stress signaling activity; acquire lapatinib resistance; and metastasize to the brain (Fig. 8t).

## Discussion

It is well-documented that targeted therapy resistance of HER2-positive breast cancer strongly associates with the onset of brain metastasis [38]. Recent work has also reported that stromal fibroblast reprogramming by SNAI2 drives solid tumor progression and upregulated PEAK1 expression [39]. In this regard, we demonstrate that high PEAK1 expression in HER2-positive breast cancer patient tissues predicts increased disease relapse (Fig. 1b) in HER2-positive breast cancers high in SNAI2 expression (Fig. 1s and Supplementary Fig. 3a) and mesenchymal stem cell (MSC) content (Fig. 2c). Notably, we also demonstrate that HER2-positive breast cancers contain fibroblastic stromal cells positive for both cytoplasmic PEAK1 and nuclear SNAI2 (Fig. 1l, m).

Marusyk and colleagues previously demonstrated that co-culturing or xenografting stromal fibroblasts together with HER2-positive breast cancer cells sustains Akt phosphorylation in the presence of lapatinib treatment [5]. More recently, Zervantonakis et al. reported that fibroblast-tumor cell signaling limits lapatinib treatment via the secretion of soluble factors which activate MTOR and antiapoptotic pathways across bulk tumor cells [13]. These results are also consistent with previous reports that MCL1 confers protection of HER2-positive breast cancer to environmental stress [40]. In this regard, we identify a new PEAK1-INHBA/activin-A-dependent axis in MSCs that is necessary for MSC-induced lapatinib resistance in HER2-positive breast cancer cells (Fig. 7). We further determine that PEAK1-expressing MSCs promote the emergence of p-Akt^high^/p-γH2AX^low^, MCL1^high^/p-γH2AX^low^ and GRP78^high^/VIM^high^ subpopulations within HER2-positive breast cancer cells that display resistance to lapatinib and are capable of enhancing tumorigenesis in vitro and in vivo (Figs. 4–6 and 8). Thus, it will be instructive to test combinatorial inhibition of INHBA/activin-A, PI3KCA/AKT1, MCL1 and GRP78 (BiP) signaling as a means to overcome HER2-targeted therapy responses.

A role for MSCs in the breast cancer microenvironment as effectors of tumor growth and metastasis has been previously established [6, 41, 42]. Notably, we observed that MSC expression of PEAK1 was required for MSCs to induce metastatic spread of HER2-positive breast cancer cells to the brain in animals treated with lapatinib (Fig. 4g). We also noted that the primary tumors in these animals showed elevated stromal αSMA expression (Fig. 4c) and no appearance of lapatinib-induced cytotoxicity (Fig. 4f). It is notable, however, that MSCs did not function to promote breast cancer metastasis (Fig. 3d and Supplementary Fig. 3c) in our analyses. This is likely due to the sensitivity of the in vivo CAM tumor model and its ability to detect the earliest stages of tumor progression [32] – stages at which the previously described pro-metastatic effects of MSCs may not have been observable. While the specific cellular processes and/or molecular machinery governing these effects will require further characterization, one possibility is that stromal expression of PEAK1 enhances tumor vascularization. This is consistent with both our observation that HER2-positive breast cancer cells xenografted with MSCs displayed increased expression of αSMA and vascular architecture in a PEAK1-dependent manner (Fig. 4c) and recent work reporting a role for PEAK1 during developmental angiogenesis [43].

Previous analyses of cell line xenografts in mice and patient tumor tissue revealed that lapatinib treatment leads to a decreased distance between αSMA-positive stromal fibroblasts and proliferating HER2-positive breast cancer cells [5], implicating juxtacrine and/or distance-dependent paracrine signaling mechanisms such as those used by morphogens. We observed that PEAK1-dependent MSC-induced protection of HER2-positive breast cancer cells against lapatinib could occur in vivo (Fig. 4) and in vitro (Figs. 5 and 6). The possibility that these MSC-driven cytoprotective effects require one or more secreted factors is supported by our identification of six secreted/soluble proteins (i.e., TGFB3, VEGFA, CSF1, CCL4, INHBA and GDF5) that were expressed by MSCs in a PEAK1-dependent manner (Fig. 7a, b). While the mechanisms by which PEAK1 regulates the expression/secretion of these factors remain to be determined, our data demonstrating that these MSC cytoprotective effects can be reversed by antagonism of activin-A in the MSC conditioned media (Fig. 7f, g) suggest that activin-A inhibition in HER2-positive breast cancers is a viable means for overcoming targeted therapy resistance. It is also notable that activins have well-established morphogen roles during normal development [44], and that previous work has reported increased INHBA/activin-A activity at the leading edge of HER2-positive breast tumors [45] and that follistatin (Fig. 7f) can suppress HER2-positive breast cancer metastasis [46]. These results together with our findings that PEAK1 expression predicts low median overall survival in breast cancer patients with high INHBA transcript levels and enriched for MSC content (Supplementary Fig. 8c), further support a role for PEAK1-dependent INHBA/activin-A expression as a mechanism by which stromal MSCs support HER2-positive breast cancer progression and therapy resistance. These studies establish a critical PEAK1-INHBA/activin-A stromal cell axis as a regulatory node that works in concert with SNAI2 to promote therapy resistance, metastasis and poor patient outcomes.

## Supplementary information

Supplementary materials

Supplementary materials
