# Supplementary material for: A SNAI2-PEAK1-INHBA stromal axis drives progression and lapatinib resistance in HER2-positive breast cancer by supporting subpopulations of tumor cells positive for antiapoptotic and stress signaling markers
Source: Oncogene. 2021 Jul 8;40(33):5224–35. doi: 10.1038/s41388-021-01906-2 (PMC8376636; doi:10.1038/s41388-021-01906-2)
Supplement: Supplementary file 1 — Supplementary materials [file 41388_2021_1906_MOESM1_ESM.docx]

**SUPPLEMENTARY INFORMATION:**

A SNAI2-PEAK1-INHBA stromal axis drives progression and lapatinib resistance in HER2-positive breast cancer by supporting subpopulations of tumor cells positive for antiapoptotic and stress signaling markers

Sarkis Hamalian et al.

**SUPPLEMENTARY MATERIALS AND METHODS**

***Cell Culture.*** RAW264.7, C3H10T1/2, NIH3T3, Swiss3T3, EA.hy926, BT474 and MCF7 cells were purchased from the American Tissue Culture Collection (ATCC). Patient-derived cancer-associated fibroblasts (CAFs) (i.e., TB123, TB125, TB98, TB130, TB122, TB129) were provided by Dr. Julia Tchou’s laboratory of the Perleman Center for Advance Medicine at the University of Pennsylvania. Isolation and maintenance protocols of these CAFs have been described [30]. Py230 cells were provided by Dr. Lesley Ellies’ laboratory at University of California San Diego. BT474-H2BeGFP and AR22 cells were provided by Dr. Joan Brugge’s laboratory at the Ludwig Center for Cancer Research & Department of Cell Biology at Harvard Medical School. Additional details about cell culture conditions can be found within the supplemental materials and methods section. RAW264.7, C3H10T1/2, NIH3T3, Swiss3T3, EA.hy926 and AR22 cells were cultured in Dulbecco’s Modified Eagle’s Medium (DMEM)/High glucose growth media supplemented with 10% fetal bovine serum (FBS), 1% penicillin/streptomycin and 0.1% gentamycin. MCF7 cells were cultured in DMEM High glucose growth media supplemented with 10% fetal bovine serum (FBS), 1% penicillin/streptomycin and 0.1% gentamycin and 0.01 mg/ml human recombinant insulin. BT474 cells were cultured in Rosewell Park Memorial Institute (RPMI-1640) growth media supplemented with 10% FBS, 1% penicillin/streptomycin and 0.1% gentamycin. CAF lines were cultured in (DMEM)/High glucose growth media supplemented with 20% fetal bovine serum (FBS), 1% penicillin/streptomycin and 0.1% gentamycin. Py230 cells were cultured in F-12K growth media supplemented with 5% fetal clone serum (FCS), 1% penicillin/streptomycin, 0.1% gentamycin and 0.1% MITO+ Serum Extender. Cultures were maintained at 37ºC with 5% CO_2_.

***Bioinformatics.*** Target gene/protein transcript levels in normal and breast cancer stroma or bulk breast cancer tissues were extracted from microarray or sequencing data published by Karnoub et al. 2006 Cell, Finak et al. 2008 Nature Medicine, Pereira et al. 2012 Nature, Nagy et al. 2018 Scientific Reports and/or Gyorffy et al. 2010 Breast Cancer Research and Treatment via the Oncomine (https://www.oncomine.org/), Cancer BioPortal (https://www.cbioportal.org/), or KMPlot (https://kmplot.com/analysis/) databases. Similarity matrices were generated using the Morpheus (https://software.broadinstitute.org/morpheus/). Immunohistochemical protein data from patient breast tumors are available in the Human Protein Atlas (http://www.proteinatlas.org). Interactome networks and gene set enrichments (GSEs) were generated using the Cytoscape Agilent Literature Search plugin (http://apps.cytoscape.org/apps/agilentliteraturesearch) and Database for Annotation, Visualization and Integrated Discovery (DAVID) platforms [31]. Data were further analyzed using IPA (QIAGEN Inc., https://www.qiagenbioinformatics.com/products/ingenuitypathway-analysis).

***Immunohistochemistry.*** The tissue was dehydrated and incubated in primary antibodies at dilution of 1:200 (i.e., αSMA, CDH1, SNAI2, and/or PEAK1) overnight. Tissues were washed and HRP-conjugated or fluorophore-conjugated secondary antibody was added to the tissue according the Vectastain Elite ABC-HRP Rabbit IgG kit protocol. The tissue was washed and the Vectastain reagent was added. In the case of HRP-based IHC, following the Vectastain reagent, the tissue was subjected to horseradish peroxidase until a noticeable color change was achieved. The tissue was then counterstained with hematoxylin, dehydrated and mounted using Permount. Peroxidase-stained tissue slides were imaged using a Zeiss microscope at 10X magnification. Fluorescence data were collected on either a Leica DMi8-based Thunder Imager with 3D deconvolution at 63X or Leica DMi6000B at 20X. Additionally, images across at least three patients per analysis group were captured and analyzed using the Human Protein Atlas [32]. TMA imaging data were analyzed for stromal expression of PEAK1 and SNAI2 via a doubly blinded method using a -2 to +2 expression scale.

***Western Blot.*** Whole cell extracts (i.e., cell lysates) were collected in standard radioimmunoprecipitation assay (RIPA) buffer containing phosphatase and protease inhibitors and rotated at 4°C overnight before pelleting insoluble material and protein analysis of supernatants. Lysate protein concentrations were determined by Bradford assay or bicinchoninic acid (BCA) assay. Proteins in lysates, normalized for 20 ug of protein/well, were reduced in 4X LDS sample buffer with DTT and separated using 4-12% Bis-Tris NuPage gels. Gels were blotted onto nitrocellulose membranes and probed for each antigen using the following antibodies at the indicated dilutions: Human PEAK1 (Millipore 1:400), Mouse PEAK1 (Abgent 1:400), α-tubulin (Pro-Sci 1:1000), GAPDH (Pro-Sci 1:1000) and β–actin (Pro-Sci 1:1000). Secondary antibodies were used at a 1:5000-1:10,000 dilution. Developed autoradiography films were scanned and analyzed for relative band intensities using Fiji software after image thresholding.

***Immunocytochemistry.*** Indicated cells fixed using 4% paraformaldehyde for 20 minutes, permeabilized for 10 minutes in 0.1% Triton-X100, blocked in 10% BSA and stained with the indicated primary/secondary antibodies at 1:400 in 2% BSA in PBS. Images were collected using a Leica DMi6000 inverted microscope at 20X magnification or Leica SP5 TCS II Confocal at 100X magnification.

***Chorioallantoic Membrane Assay (CAM Assay).*** Rhode Island Red Hatching Eggs were purchased from Meyer Hatchery and incubated at 38 degrees Celsius and 60% humidity for 10 days on an automated egg turner. On day 10, the shell was sterilized with Rocadyne (RMC) and a small hole was made at the blunt end of the egg to evacuate the air pocket and pull the CAM away from the shell and shell membrane. While candeling the egg, a small rectangular window was cut over the umbilical vein using a dremel (Black and Decker). A sterile silicon ring was placed onto the CAM in an area of dense vasculature. Cells were trypsinized, counted and resuspended in ice-cold 100% GFR Matrigel using ice-cold pipette tips at the following specifications: mono-xenografts had 1e6 cells suspended in 40µl of Matrigel per embryo and co-xenografts of breast cancer cells with stromal cells had 1e6 cells per line suspended in 40µl of Matrigel per embryo. Xenografts and co-xenograft suspensions were then applied to the CAM using ice-cold pipette tips. Sterile surgical tape was applied to the windowed area as well as the blunt end and the eggs were placed back into the incubator without egg turning. In experiments with drug treatments, two days after xenografting, the eggs were re-sterilized and the surgical tape was carefully peeled back to reveal one corner of the window. Drugs are prepared at 2X the final concentration (i.e., 3600 nM or 2 µM lapatinib) in 40 uL of ice-cold 100% GFR Matrigel per embryo. At day 17, the embryo was then extracted from the egg and the developing chicken sacrificed via decapitation. The primary CAM tumor was dissected, brain and lung tissues were weighed and divided into two samples each for flash-freezing (in an EtOH/dry ice bath) and fixing (in 10% Formalin), respectively.

For qPCR analysis of relative metastasis, 20mg of relevant tissues were weighed, homogenized using a micro-homegenizer (ClaremontBio Solutions) in digestion solution and then heated at 56˚C for 3.5 hours. Genomic DNA was then purified using GeneJet Genomic DNA purification kit (ThermoFisher Scientific) and the concentration measured by NanoDrop. gDNA was stored at -20˚C for downstream qPCR analysis. Samples of gDNA were diluted with nuclease free water so that addition of 11.25 µl of this diluted sample delivers 56.25 ng of gDNA. Primers were purchased from Integrated DNA for human Alu repeat (sense: 5′ ACG CCT GTA ATC CCA GCA CTT 3′ and antisense: 5′ TCG CCC AGG CTG GAG TGC A 3′) and chicken gapdh (sense: 5′ GAG GAA AGG TCG CCT GGT GGA TCG; antisense: 5′ GGT GAG GAC AAG CAG TGA GGA ACG) and used at a concentration of 10nmol/mL per reaction. A master mix was made so that each well would contain 12.5 μL of Maxima SYBR® Green (Thermo Scientific) and 1.25 µLl of gene-specific primer. Contents for experimental wells include 11.25 µL diluted gDNA and 13.75 µL of master mix. qPCR plates were processed using the ABI7300 instrument with the following thermal cycles settings: Stage 1, 1 repetition - 50°C for 2 minutes / Stage 2, 1 repetition - 95°C for 30 seconds / Stage 3, 40 repetitions at 95°C for 15 seconds / Stage 4 at 62°C for 1 minute. Relative metastasis was calculated as previously described [33]. Briefly, dCt values were calculated as aluCt - gapdhCt. ddCt values were calculated as dCt_variable_ - Average dCt_control_. RQ

values were calculated as 2e^(−ddCt)^.

To calculate the number of animals needed, each treatment group or control group will require typically 10 chicken embryos (unless otherwise specified) multiplied by the number of sampling dates (i.e., one), number of treatment/control groups (i.e., 12), and/or endpoint assays (i.e., one). Based upon an anticipated growth benefit of 2-fold in the sample group (i.e., MSC-containing xenografts), and in order to achieve statistical significance of p < 0.05 (Student’s t-test). No randomization or blinding was used in these animal studies.

***Cell Proliferation/Viability Assay.*** Cells were plated at 1e3 cells/well (200µL) in a 96-well plate and allowed to attach overnight. Cells were then treated with conditioned media from stromal cells for 72 hours. For experiments with drug treatment, drugs were added 24 hours after CM treatment. At treatment endpoint, 40µL of the CellTiter 96® AQueous One Solution (Promega) was added to each well. Absorbance readings were measured at 490nm after 3 hours of incubation with reagent using a Spectra Max 190 (Molecular Devices). Absorbance readings are directly proportional to the number of viable cells.

***IncuCyte.*** Cells were trypsinized, pelleted, and resuspended in RPMI medium supplemented with 2% FBS before plating in 200 uL/well into 96-well plates. Mono-cultures received 5e4 cells of cells. Co-cultures received 2.5e4 cells of each respective cell type per well. Two days after seeding, drugs or vehicle controls along with EtBr were prepared at five times the target concentration in 2% FBS-RPMI, and 50uL was added to the designated wells for treatment to obtain the final working concentrations. IncuCyte® Live Cell Analysis Imaging System was used according to manufacturer’s protocol. Four images were taken per well for both the green and red channels. These were collected every 3 hours for the indicated time from time of plating up to 6 days after drug treatment.

***Cyclic Immunofluorescence (CycIF).*** Cyclic multiplex antigen staining and Hoescht nuclear counterstaining was carried out on paraformaldehyde (PFA)-fixed mono- or co-culture cells on ultra-optically clear, flat-bottom, black-walled 96-well plates as previously described [34]. Briefly, H2B-eGFP labelled breast cancer cells (alone or in culture with shRNA derivatives of the C3H10T1/2 MSCs) were fixed with 4% PFA for one hour, followed by nuclear Hoescht 33342 staining and 16-bit image acquisition at 20X magnification across four channels. Sample fluorophores were bleached in 4% H_2_0_2_ + 20 mM HCl via exposure to UV light for 3 hours and restained with the subsequent round of antigen-specific antibodies overnight at 4^o^C. Subsequent cycles of nuclear staining, imaging, bleaching and restaining were performed as needed. Fluorescently complementary antibody sets were selected from previously validated lists (see <https://www.cycif.org/>) for the following antigens: p-Akt (CST #4071S, clone D9E), MCL1 (CST #94296, clone D2W9E), p-γH2AX (Biolegend #613407, clone 2F3), GRP78 (Abcam #115638, clone 10C3), VIM (CST #5741, clone D21H3), αSMA (Invitrogen #41976082, clone 1A4), p65NFκB (Abcam #190589, clone E379). Image registration and nuclear/cytoplasmic antigen integrated signal density was performed using ImageJ StackReg and MultiStackReg plugins together with previously reported macro scripts [34] modified for experiment specific well-plate layout and cycle number parameters. Dimensionality reduction and subsequent analysis of single-cell data was performed using the t-distributed stochastic neighbor embedding (tSNE) package within the FlowJo SegGeq application (concatenated single-cell antigen data are included as Dataset 2, Learning Configuration set to Auto – optSNE with Exact KNN algorithm, Barnes-Hut GradientDescent algorithm, 1000 iterations, perplexity = 30 and learning rate = 3239).

**SUPPLEMENTARY FIGURE LEGENDS**

**Supplementary Figure 1:** Kaplan-Meier overall survival (OS, top) and distant metastasis-free survival (DMFS, bottom) curves for low or high transcript levels of in all (left) (OS n = 1880, DMFS n = 2767) and HER2-positive (right) breast cancer patients (OS n = 223, DMFS n = 260).

**Supplementary Figure 2:** a. Similarity matrix of average transcript levels for indicated genes in breast cancer stroma from patients categorized as having poor (left, n = 8), mixed (center, n = 27) or good (right, n = 17) outcomes. b. Representative IHC/IF widefield microscopy images of nuclear (DAPI), PEAK1, SNAI2 and CDH1 stains in HER2-negative (top) and HER2-positive (bottom) breast cancers.

**Suplementary Figure 3:** a-c. Kaplan-Meier overall survival (OS), relapse free survival (RFS) and/or distant metastasis free survival (DMFS) curves for patients with low or high SNAI2 (a), MYC (b) or FN1 (c) transcript levels across all breast cancer subtypes (OS n = 1880, RFS n = 4934, DMFS n = 2767) or HER2-positive breast cancers (OS n = 223, RFS n = 461, DMFS n = 260).

**Supplementary Figure 4:** a-b. Representative immunofluorescence widefield microscopy images for nuclear (DAPI), actin (Phalloidin) and PEAK1 in cancer associated fibroblasts (CAFs) isolated from TB98 HER2-negative (top) and TB129 HER2-positive (bottom) breast cancer patient biopsies.

**Supplementary Figure 5:** a. Assay endpoint mages of indicated tissues for MCF7 cells, MCF7 cells + TB130 CAFs or MCF7 cells pre-treated (72 hours) with TB130 conditioned media and xenografted with TB130 CAFs (MCF7 + PT-TB130). b. Quantified primary tumor mass of experiment in (a). c. Relative metastasis of MCF7 cells in the lung (left) and brain (right) of experiment in (a). * indicates a p-value < 0.05 as determined by a One-Way ANOVA w/ multiple comparisons test.

**Supplementary Figure 6:** a. Representative phase and phase+GFP overlay images for the indicated mono-culture or co-culture BT474 cells with either C3H10T1/2 MSCs or AR22 breast fibroblasts. b. Time-course traces for cell number measured by GFP signal (left) and cell death measured by EtBr signal (right) across all mono- or co-culture cell combinations and lapatinib treatment doses.

**Supplementary Figure 7:** a. Cell viability analysis of MCF7 cells treated with mock or TB130 CM. b. Cell viability analysis of mouse Py230 cells grown in serum-free or complete media and treated with mock CM or CM from the indicated shRNA derivatives of C3H10T1/2 cells. *, ***, or **** indicates a p-value < 0.05, 0.001 or 0.0001, respectively, as determined by a One-Way or Two-Way ANOVA w/ multiple comparisons post-test.

**Supplemental Figure 8:** a. Kaplan-Meier Relapse-Free Survival (RFS) curves of breast cancer patients across all subtypes in relation to low or high expression of the indicated cytokines (n = 3951). b. Kaplan-Meier RFS curves of breast cancer patients with HER2-positive disease in relation to low or high expression of the indicated cytokines (n = 252). c. Kaplan-Meier Overall Survival (OS) curves for high and low PEAK1 in patients enriched for MSC content and selected for high expression of the indicated cytokine (n = 380, 392, 382, 12, 350 and 382, respectively).

**Supplemental Figure 9:** a. t-SNE plots of the GFP-positive (top) BT474 breast cancer cells from mono-culture or C3HshScr, C3HshP1(1) or C3HshP1(3) co-culture conditions or GFP-negative (bottom) MSCs from BT474-C3H shRNA derivative co-cultures overlayed with cell populations corresponding to control or lapatinib treatment conditions. b. Averaged GFP-negative MSC number at 48 hours post-therapy treatment and quantification of area under the curve (AUC) for each cell culture condition across treatment conditions. c-f. Average integrated signal intensity for each of five antigen markers in BT474 monoculture vs. BT474+C3HshScr co-culture conditions (x-axis labels) for the four identified GFP-positive breast cancer cell subpopulations (c, d, e, and f) gated for low/high (left/right graphs) expression of the complementary sixth antigen marker.

**Supplemental Figure 10:** Cytoscape Agilent Literature Search interactome with associated two-dimensional enriched ontology plots for the gene set search of SNAI2, PEAK1, INHBA, CCL4, GDF5, MCL1, AKT1, H2AFX, GRP78 and VIM.
