# Supplementary material for: A SNAI2-PEAK1-INHBA stromal axis drives progression and lapatinib resistance in HER2-positive breast cancer by supporting subpopulations of tumor cells positive for antiapoptotic and stress signaling markers
Source: Oncogene. 2021 Jul 8;40(33):5224–35. doi: 10.1038/s41388-021-01906-2 (PMC8376636; doi:10.1038/s41388-021-01906-2)
Supplement: Supplementary file 2 — Supplementary materials [file 41388_2021_1906_MOESM2_ESM.pdf]

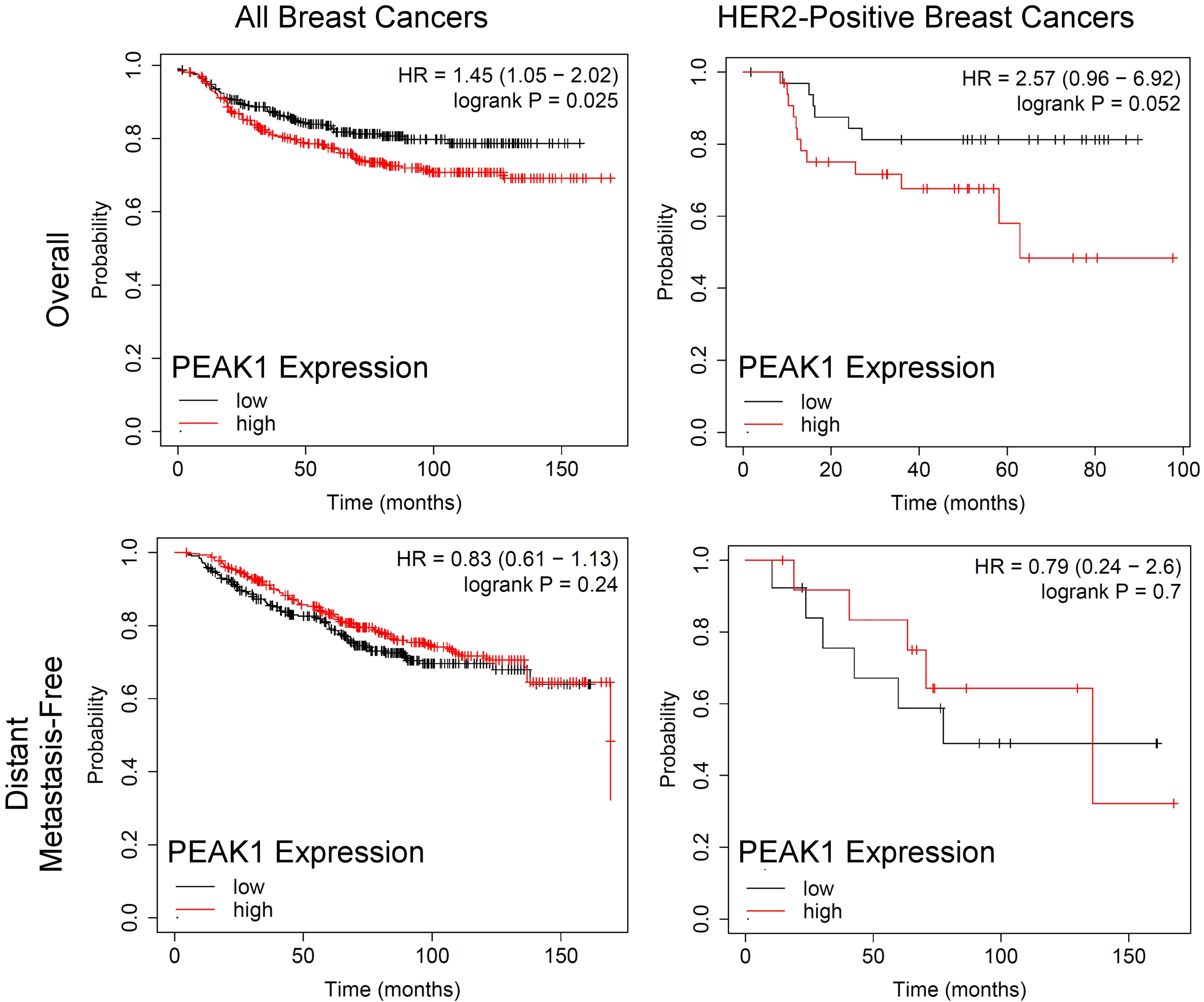

All Breast Cancers
HER2-Positive Breast Cancers
Overall
Distant Metastasis-Free
HR = 1.45 (1.05 − 2.02)
logrank P = 0.025
HR = 2.57 (0.96 − 6.92)
logrank P = 0.052
HR = 0.83 (0.61 − 1.13)
logrank P = 0.24
HR = 0.79 (0.24 − 2.6)
logrank P = 0.7
PEAK1 Expression
low
high
Probability
Time (months)

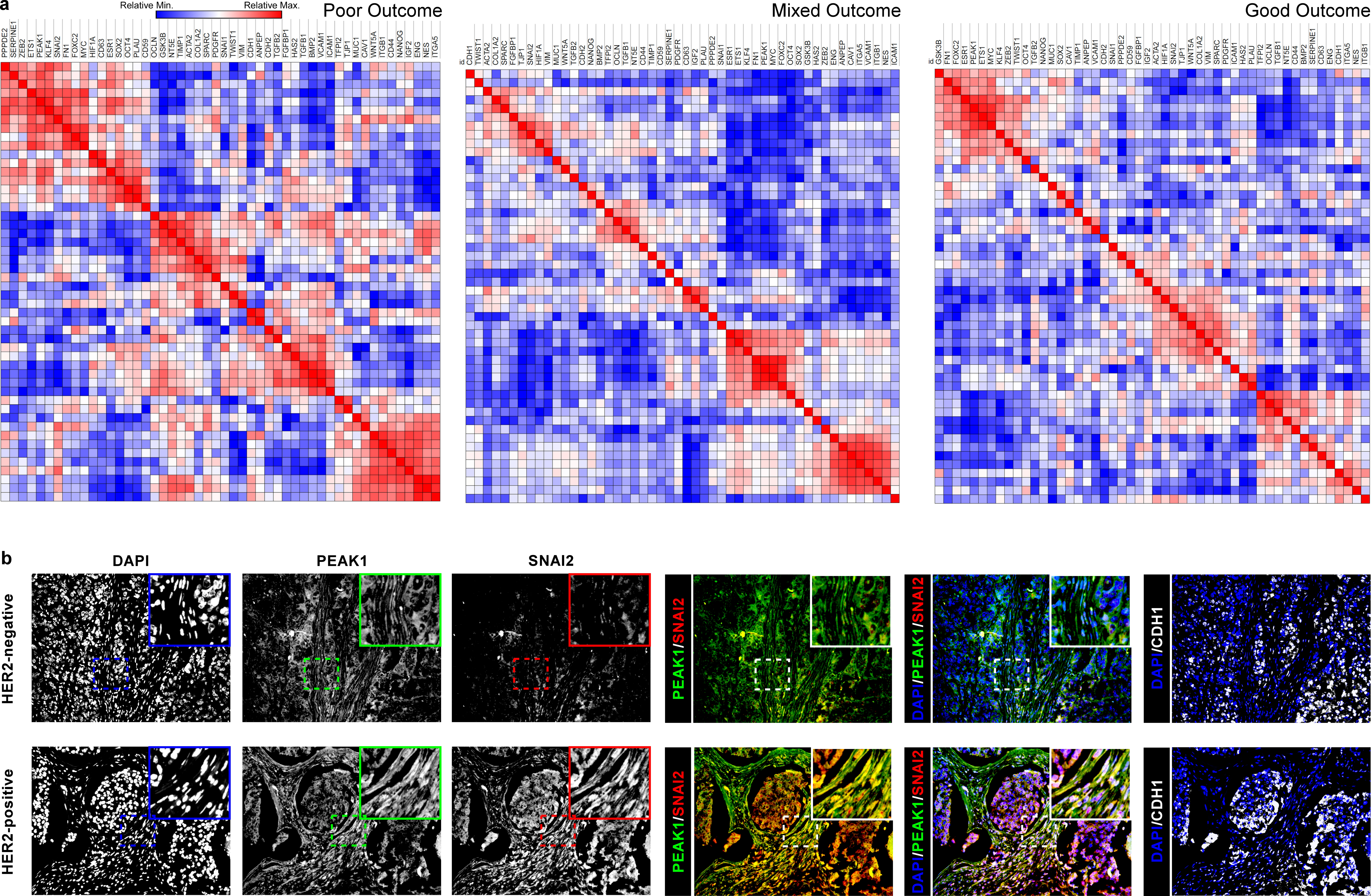
a
Relative Min.
Relative Max.
Poor Outcome
PPPDE2 SERPINE1 ZEB2 ETS1 PEAK1 KLF4 SNAI2 FN1 FOXC2 MYC HIF1A CD63 ESR1 SOX2 OCT4 PLAU CD59 OCLN GSK3B NT5E TIMP1 ACTA2 COL1A2 SPARC PDGFR SNAI1 TWIST1 VIM CDH1 ANPEP CDH2 TGFB2 FGFBP1 HAS2 TGFB1 BMP2 VCAM1 ICAM1 TFPI2 TJP1 MUC1 CAV1 WNT5A ITGB1 CD44 NANOG IGF2 ENG NES ITGA5
Mixed Outcome
CDH1 TWIST1 ACTA2 COL1A2 SPARC FGFBP1 TJP1 SNAI2 HIF1A VIM MUC1 WNT5A TGFB2 CDH2 NANOG BMP2 TFPI2 OCLN TGFB1 NT5E CD44 TIMP1 CD59 SERPINE1 PDGFR CD63 IGF2 PLAU PPPDE2 SNAI1 ESR1 ETS1 KLF4 FN1 PEAK1 MYC FOXC2 OCT4 SOX2 GSK3B HAS2 ZEB2 ENG ANPEP CAV1 ITGA5 VCAM1 ITGB1 NES ICAM1
Good Outcome
GSK3B FN1 FOXC2 ESR1 PEAK1 ETS1 MYC KLF4 ZEB2 TWIST1 OCT4 TGFB2 NANOG MUC1 SOX2 CAV1 TIMP1 ANPEP VCAM1 CDH2 SNAI1 PPPDE2 CD59 FGFBP1 IGF2 ACTA2 HIF1A SNAI2 TJP1 WNT5A COL1A2 VIM SPARC PDGFR ICAM1 HAS2 PLAU TFPI2 OCLN TGFB1 NT5E CD44 BMP2 SERPINE1 CD63 ENG CDH1 ITGA5 NES ITGB1
b
DAPI
PEAK1
SNAI2
HER2-negative
HER2-positive
PEAK1/SNAI2
DAPI/PEAK1/SNAI2
DAPI/CDH1

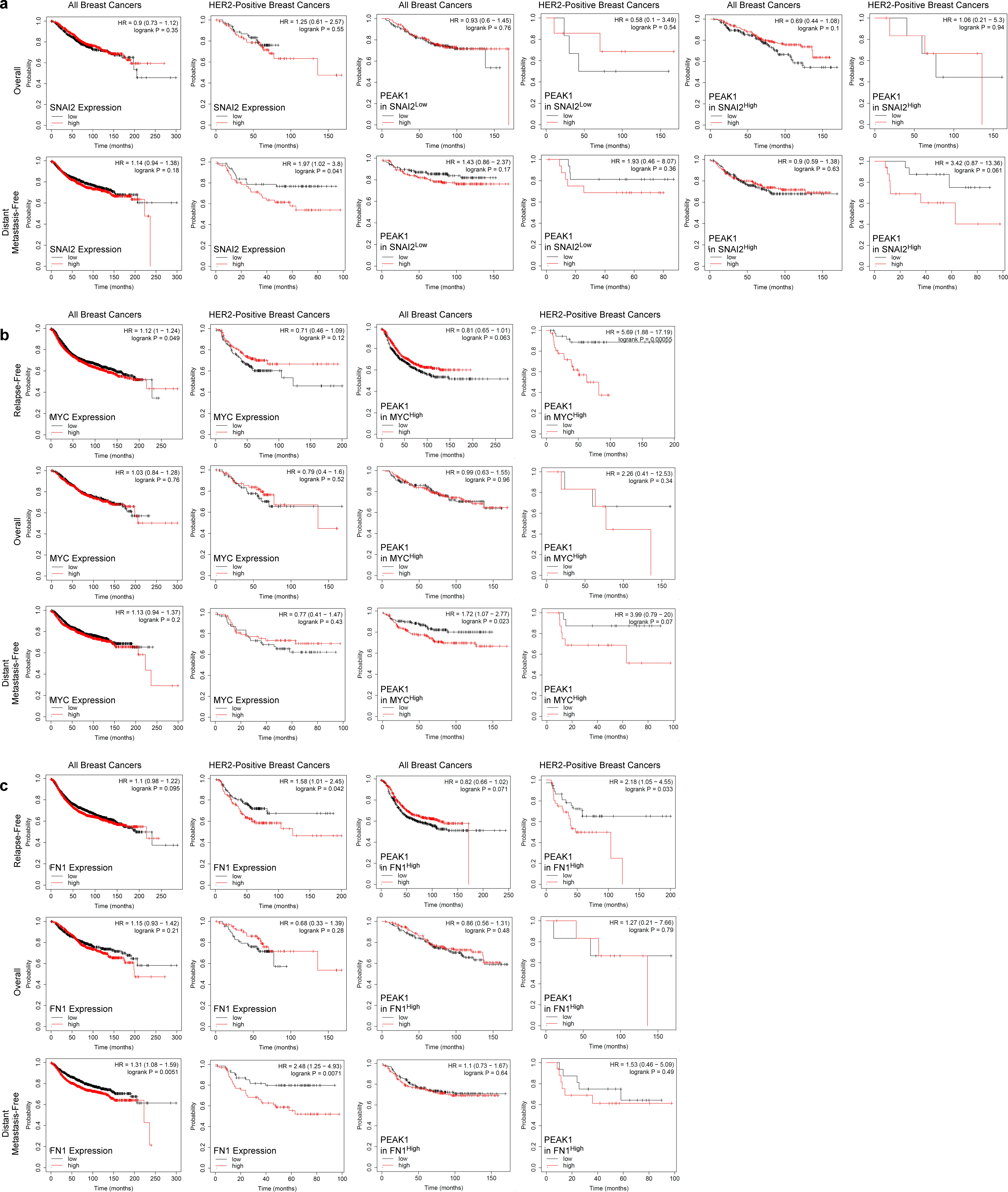

a
b
c
All Breast Cancers
HER2-Positive Breast Cancers
Overall
Distant Metastasis-Free
Relapse-Free
Probability
Time (months)
low
high
SNAI2 Expression
MYC Expression
FN1 Expression
PEAK1 in SNAI2Low
PEAK1 in SNAI2High
PEAK1 in MYCHigh
PEAK1 in FN1High
HR = 0.9 (0.73 − 1.12) logrank P = 0.35
HR = 1.25 (0.61 − 2.57) logrank P = 0.55
HR = 0.93 (0.6 − 1.45) logrank P = 0.76
HR = 0.58 (0.1 − 3.49) logrank P = 0.54
HR = 0.69 (0.44 − 1.08) logrank P = 0.1
HR = 1.06 (0.21 − 5.3) logrank P = 0.94
HR = 1.14 (0.94 − 1.38) logrank P = 0.18
HR = 1.97 (1.02 − 3.8) logrank P = 0.041
HR = 1.43 (0.86 − 2.37) logrank P = 0.17
HR = 1.93 (0.46 − 8.07) logrank P = 0.36
HR = 0.9 (0.59 − 1.38) logrank P = 0.63
HR = 3.42 (0.87 − 13.36) logrank P = 0.061
HR = 1.12 (1 − 1.24) logrank P = 0.049
HR = 0.71 (0.46 − 1.09) logrank P = 0.12
HR = 0.81 (0.65 − 1.01) logrank P = 0.063
HR = 5.69 (1.88 − 17.19) logrank P = 0.00055
HR = 1.03 (0.84 − 1.28) logrank P = 0.76
HR = 0.79 (0.4 − 1.6) logrank P = 0.52
HR = 0.99 (0.63 − 1.55) logrank P = 0.96
HR = 2.26 (0.41 − 12.53) logrank P = 0.34
HR = 1.13 (0.94 − 1.37) logrank P = 0.2
HR = 0.77 (0.41 − 1.47) logrank P = 0.43
HR = 1.72 (1.07 − 2.77) logrank P = 0.023
HR = 3.99 (0.79 − 20) logrank P = 0.07
HR = 1.1 (0.98 − 1.22) logrank P = 0.095
HR = 1.58 (1.01 − 2.45) logrank P = 0.042
HR = 0.82 (0.66 − 1.02) logrank P = 0.071
HR = 2.18 (1.05 − 4.55) logrank P = 0.033
HR = 1.15 (0.93 − 1.42) logrank P = 0.21
HR = 0.68 (0.33 − 1.39) logrank P = 0.28
HR = 0.86 (0.56 − 1.31) logrank P = 0.48
HR = 1.27 (0.21 − 7.66) logrank P = 0.79
HR = 1.31 (1.08 − 1.59) logrank P = 0.0051
HR = 2.48 (1.25 − 4.93) logrank P = 0.0071
HR = 1.1 (0.73 − 1.67) logrank P = 0.64
HR = 1.53 (0.46 − 5.09) logrank P = 0.49

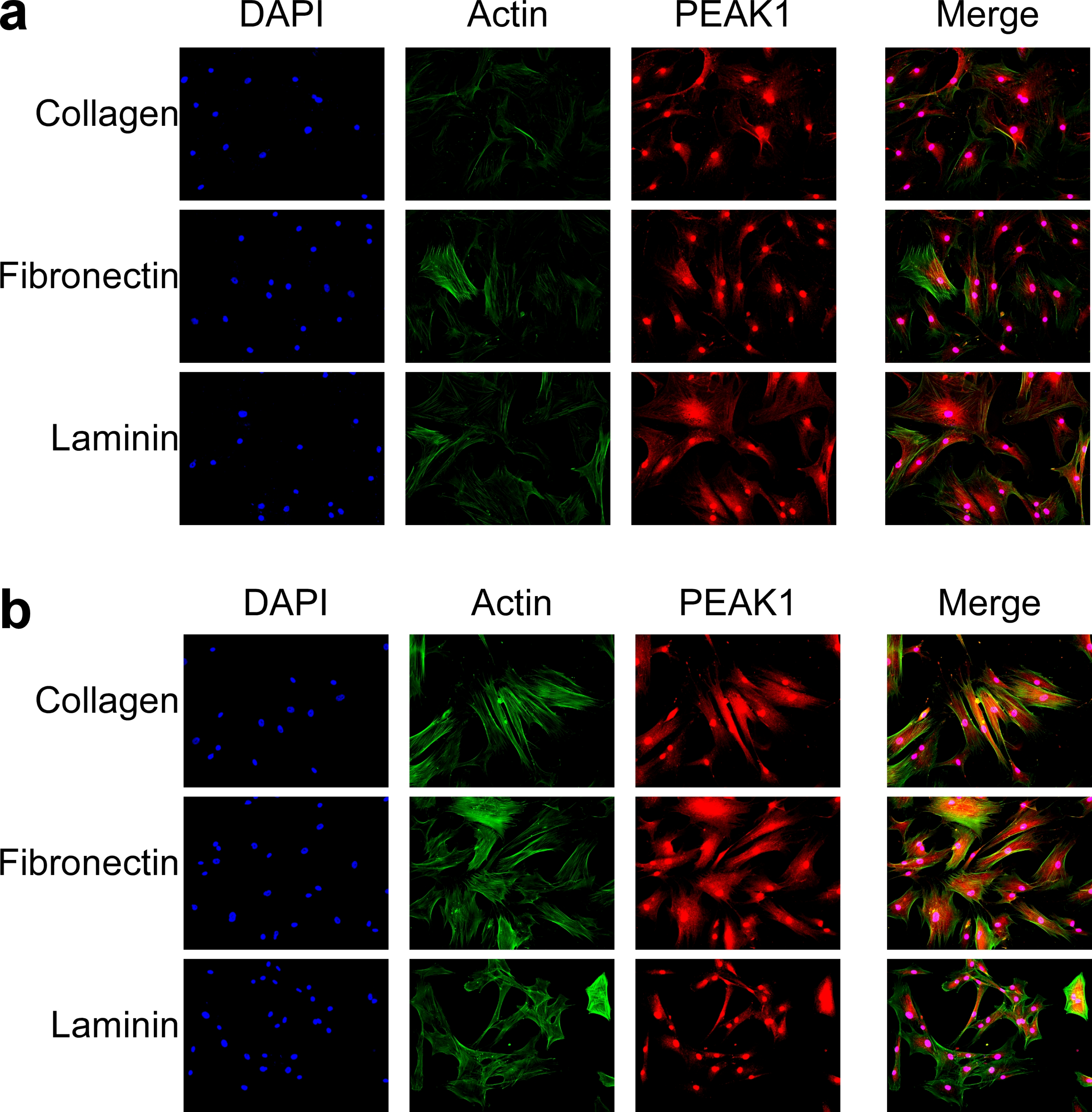
a
DAPI
Actin
PEAK1
Merge
Collagen
Fibronectin
Laminin
b
DAPI
Actin
PEAK1
Merge
Collagen
Fibronectin
Laminin

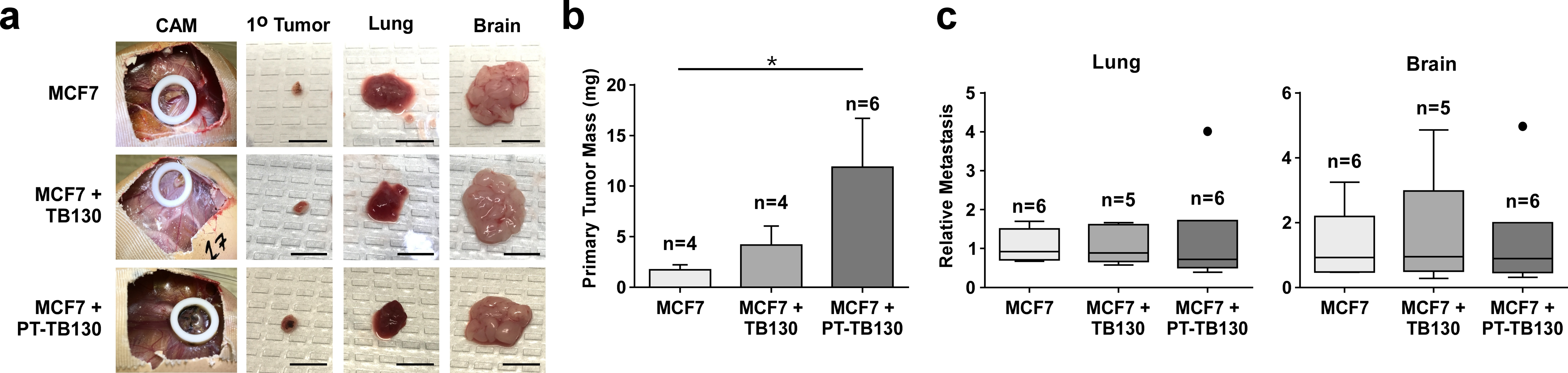

a
CAM
1o Tumor
Lung
Brain
MCF7
MCF7 + TB130
MCF7 + PT-TB130
b
Primary Tumor Mass (mg)
*
n=4
n=4
n=6
MCF7
MCF7 + TB130
MCF7 + PT-TB130
c
Lung
Brain
Relative Metastasis
n=6
n=5
n=6
n=6
n=5
n=6
MCF7
MCF7 + TB130
MCF7 + PT-TB130

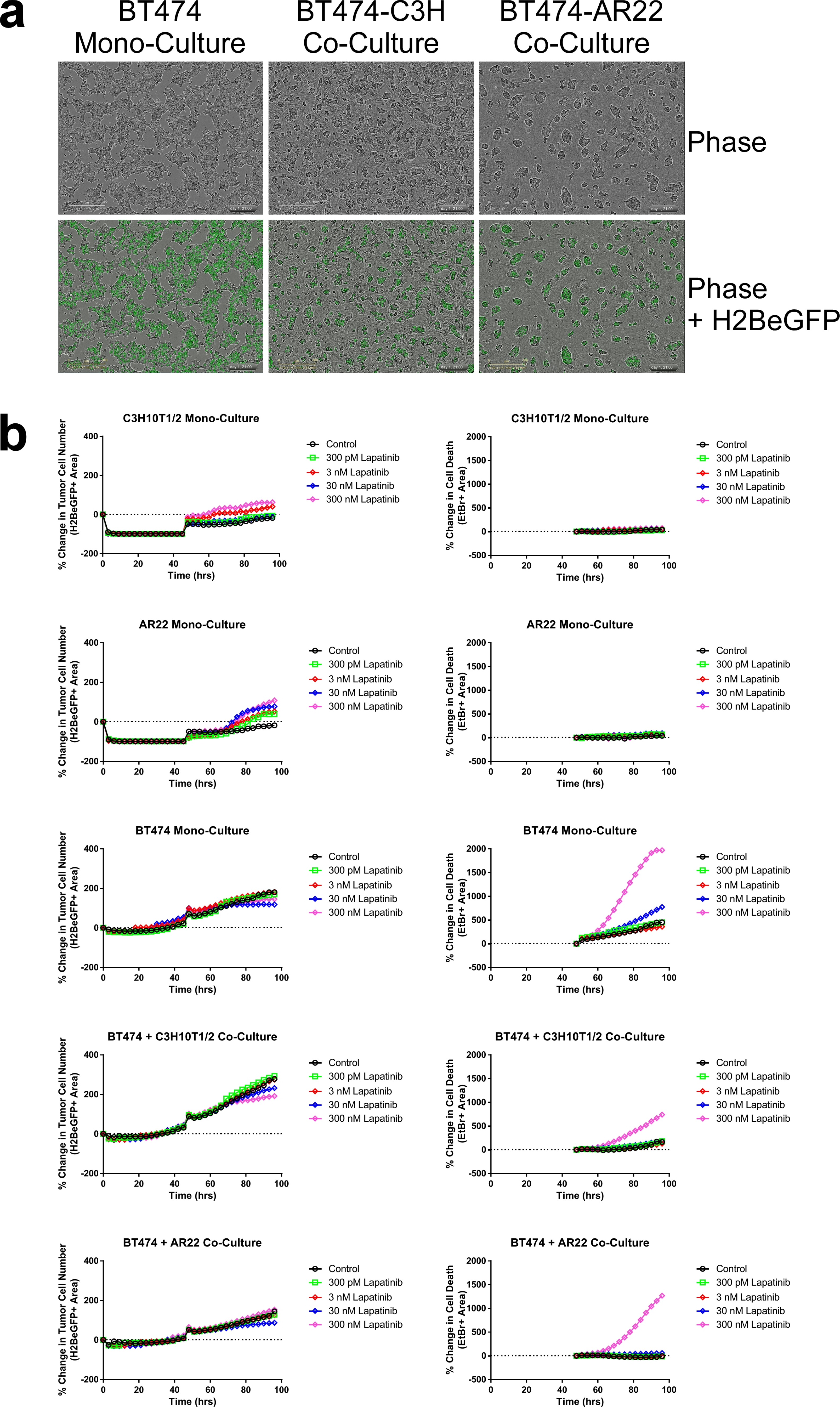
a
BT474
Mono-Culture
BT474-C3H
Co-Culture
BT474-AR22
Co-Culture
Phase
Phase
+ H2BeGFP
b
C3H10T1/2 Mono-Culture
AR22 Mono-Culture
BT474 Mono-Culture
BT474 + C3H10T1/2 Co-Culture
BT474 + AR22 Co-Culture
% Change in Tumor Cell Number
(H2BeGFP+ Area)
% Change in Cell Death
(EtBr+ Area)
Time (hrs)
Control
300 pM Lapatinib
3 nM Lapatinib
30 nM Lapatinib
300 nM Lapatinib

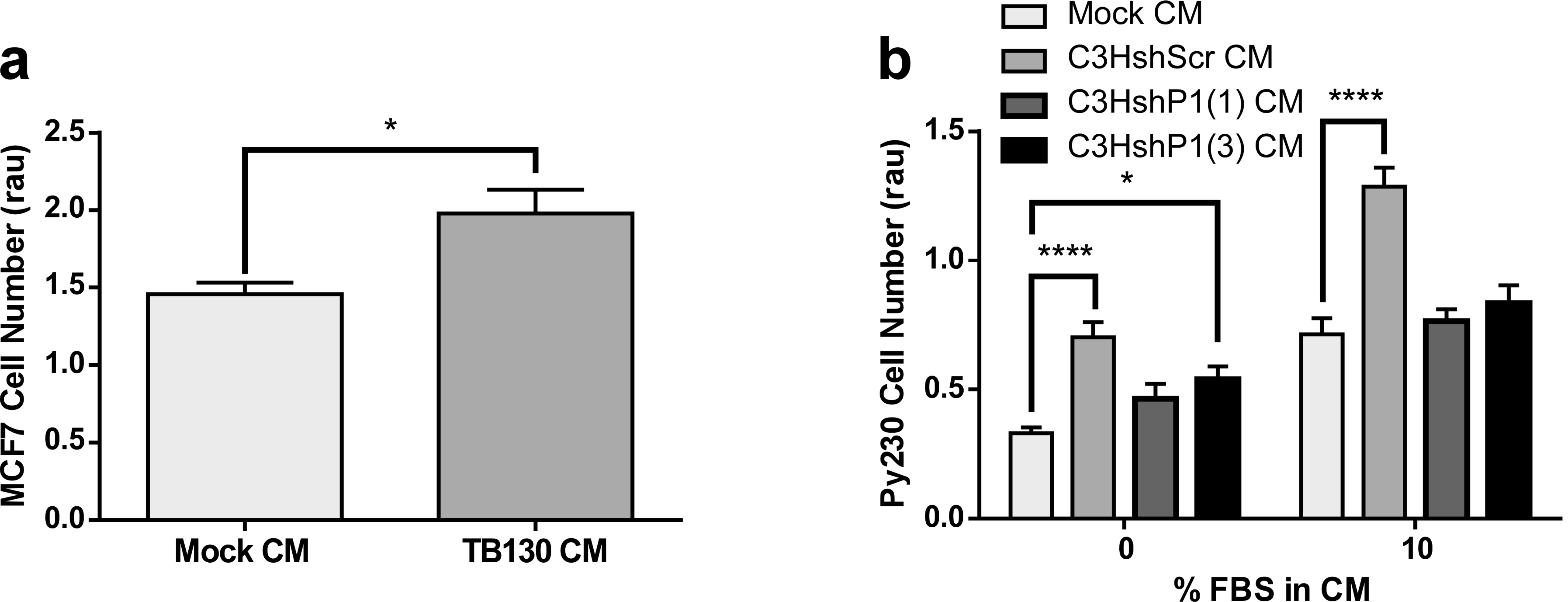

a
*
2.5
2.0
1.5
1.0
0.5
0.0
MCF7 Cell Number (rau)
Mock CM
TB130 CM
b
Mock CM
C3HshScr CM
C3HshP1(1) CM
C3HshP1(3) CM
****
*
****
1.5
1.0
0.5
0.0
Py230 Cell Number (rau)
0
10
% FBS in CM

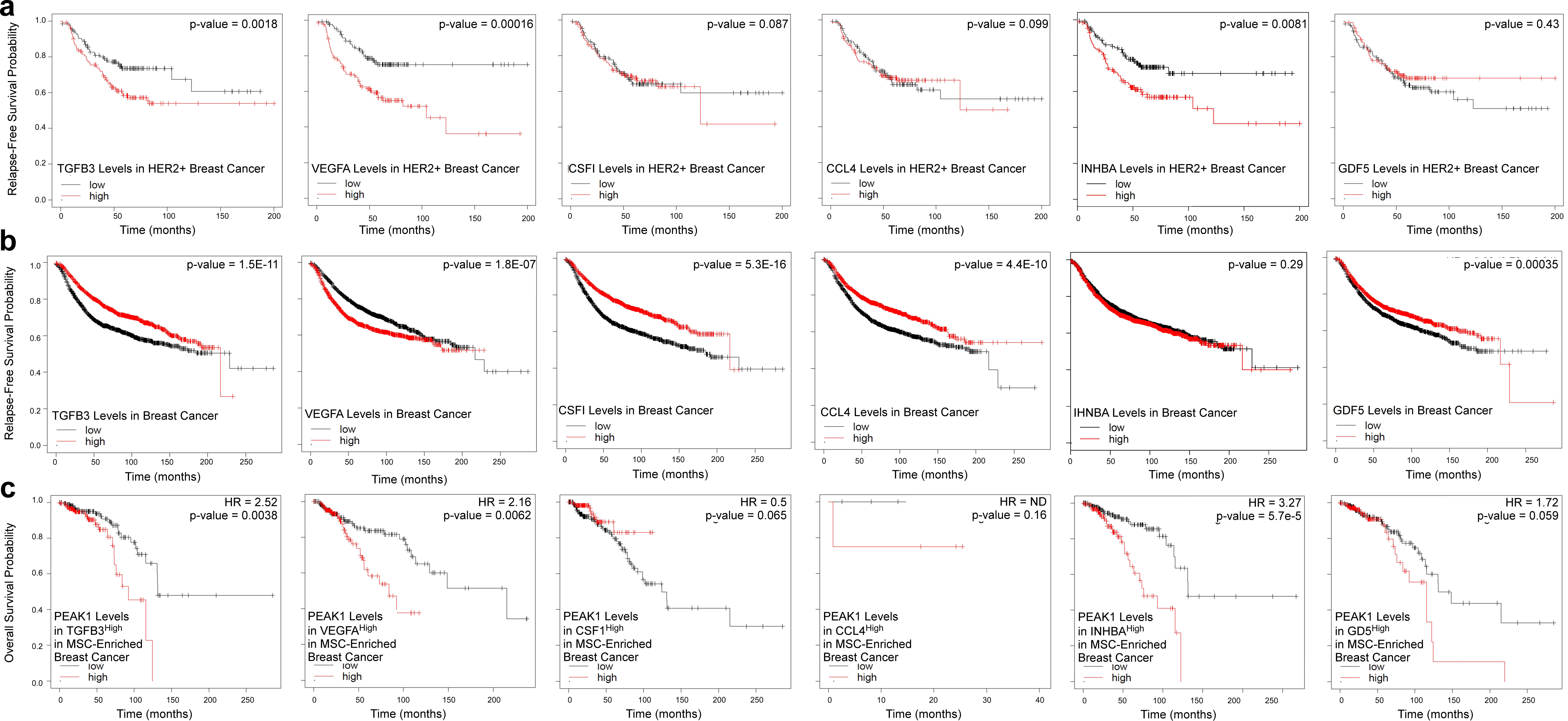

a
Relapse-Free Survival Probability
p-value = 0.0018
TGFB3 Levels in HER2+ Breast Cancer
p-value = 0.00016
VEGFA Levels in HER2+ Breast Cancer
p-value = 0.087
CSFI Levels in HER2+ Breast Cancer
p-value = 0.099
CCL4 Levels in HER2+ Breast Cancer
p-value = 0.0081
INHBA Levels in HER2+ Breast Cancer
p-value = 0.43
GDF5 Levels in HER2+ Breast Cancer
low
high
Time (months)
b
Relapse-Free Survival Probability
p-value = 1.5E-11
TGFB3 Levels in Breast Cancer
p-value = 1.8E-07
VEGFA Levels in Breast Cancer
p-value = 5.3E-16
CSFI Levels in Breast Cancer
p-value = 4.4E-10
CCL4 Levels in Breast Cancer
p-value = 0.29
IHNBA Levels in Breast Cancer
p-value = 0.00035
GDF5 Levels in Breast Cancer
low
high
Time (months)
c
Overall Survival Probability
HR = 2.52
p-value = 0.0038
PEAK1 Levels in TGFB3High in MSC-Enriched Breast Cancer
HR = 2.16
p-value = 0.0062
PEAK1 Levels in VEGFAHigh in MSC-Enriched Breast Cancer
HR = 0.5
p-value = 0.065
PEAK1 Levels in CSF1High in MSC-Enriched Breast Cancer
HR = ND
p-value = 0.16
PEAK1 Levels in CCL4High in MSC-Enriched Breast Cancer
HR = 3.27
p-value = 5.7e-5
PEAK1 Levels in INHBAHigh in MSC-Enriched Breast Cancer
HR = 1.72
p-value = 0.059
PEAK1 Levels in GD5High in MSC-Enriched Breast Cancer
low
high
Time (months)

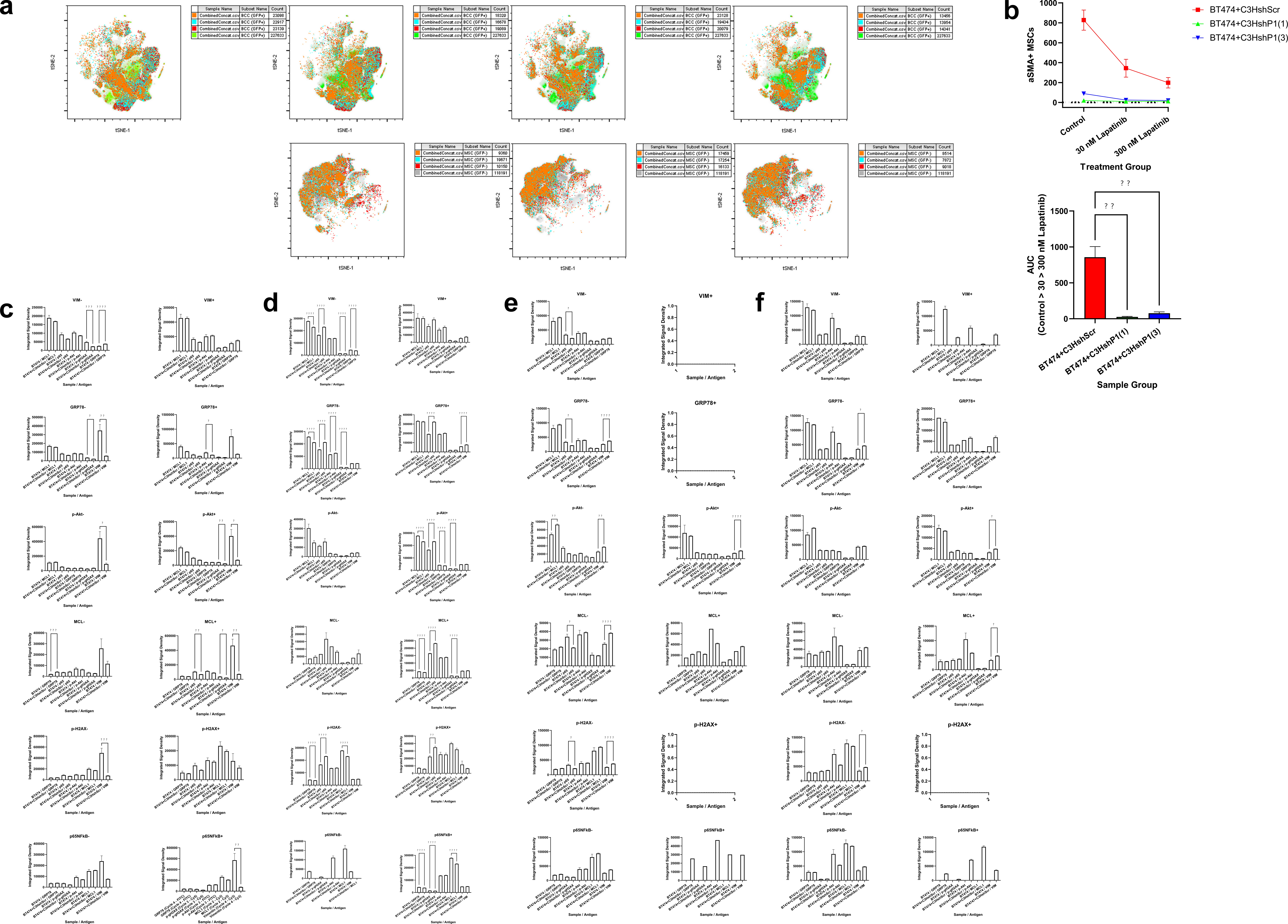
a
b
BT474+C3HshScr
BT474+C3HshP1(1)
BT474+C3HshP1(3)
aSMA+ MSCs
Control
30 nM Lapatinib
300 nM Lapatinib
Treatment Group
AUC
(Control > 30 > 300 nM Lapatinib)
Sample Group
c
d
e
f
tSNE-1
tSNE-2
Sample Name
Subset Name
Count
VIM-
VIM+
GRP78-
GRP78+
p-Akt-
p-Akt+
MCL-
MCL+
p-H2AX-
p-H2AX+
p65NFkB-
p65NFkB+
Integrated Signal Density
Sample / Antigen

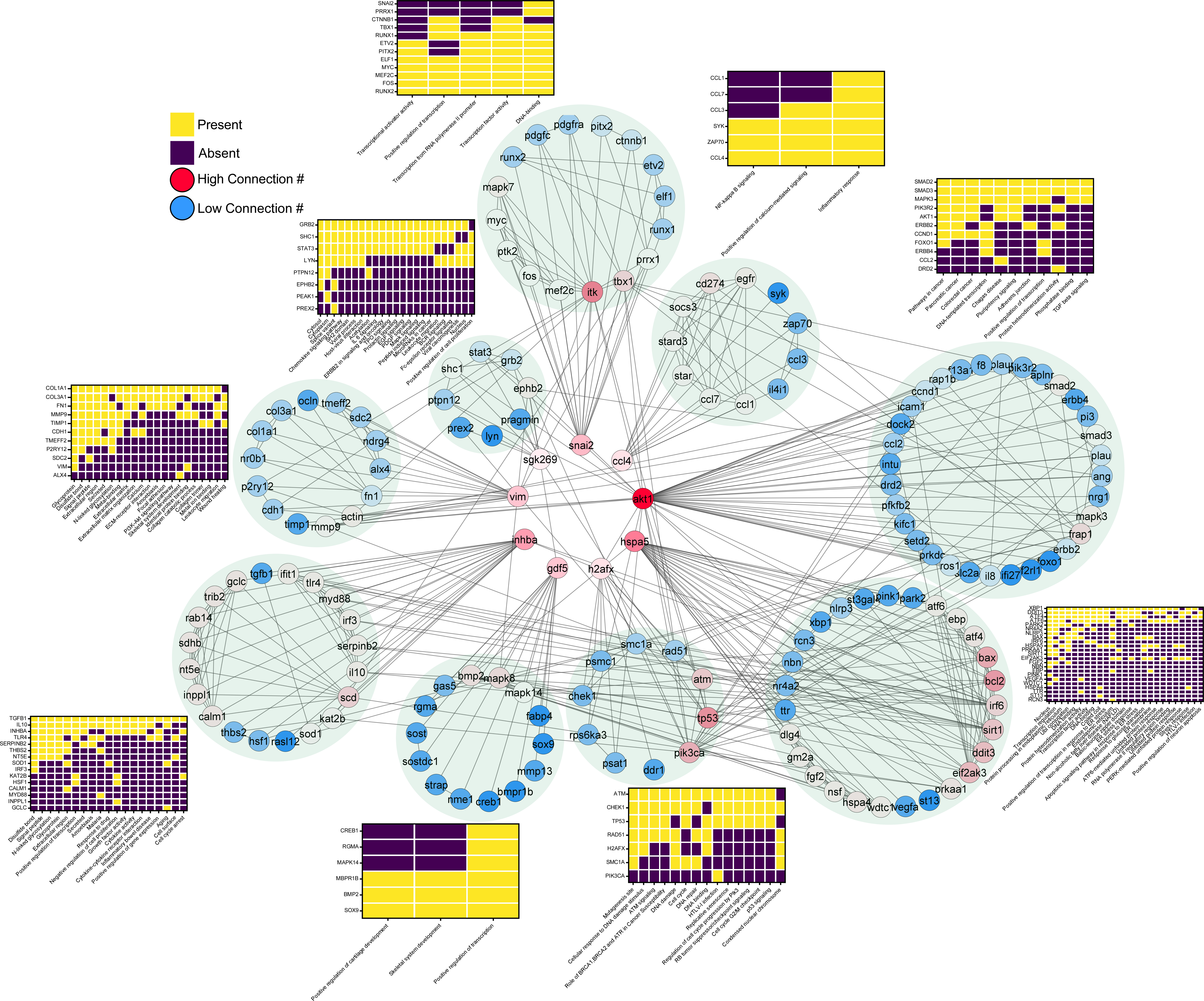

Present
Absent
High Connection #
Low Connection #
akt1
hspa5
vim
inhba
snai2
sgk269
ccl4
itk
tbx1
h2afx
gdf5
tp53
pik3ca
atm
bax
bcl2
